# Elucidation of the miR164c-Guided Gene/Protein Interaction Network Controlling Seed Vigor in Rice

**DOI:** 10.3389/fpls.2020.589005

**Published:** 2020-11-12

**Authors:** Kerui Huang, Shiqi Zhou, Kaimin Shen, Yan Zhou, Feng Wang, Xiaocheng Jiang

**Affiliations:** ^1^College of Life Sciences, Hunan Normal University, Changsha, China; ^2^Hunan Province Key Laboratory of Crop Sterile Germplasm Resource Innovation and Application, Changsha, China

**Keywords:** *Oryza sativa* L., seed vigor, miR164c, transcriptome, proteome, regulatory network

## Abstract

MicroRNAs (miRNAs) play important roles in various aspects of plant physiology and metabolism. The expression level of miR164c is negatively correlated with seed vigor in rice (*Oryza sativa* L.); however, the mechanism of seed vigor regulation by miR164c remains unknown. Anti-aging capacity is an important indicator of seed vigor. Here, we report an miR164c-guided gene/protein interaction network that regulates the anti-aging ability of rice seeds. Seeds of the wild-type (WT) rice cultivar “Kasalath” and its transgenic derivatives, miR164c-silenced line (MIM164c) and miR164c overexpression line (OE164c), with significant differences in anti-aging capacity, showed significant differences in gene and protein expression levels. The differentially expressed genes (DEGs) or proteins were significantly enriched in six metabolic functional categories related to seed vigor, including “stress response,” “protein processing in endoplasmic reticulum (ER),” “embryo development,” “serine-type endopeptidase inhibitor,” “energy metabolism,” and “other.” Differences in the expression levels of genes or proteins related to energy metabolism, serine endopeptidase, and stress response in seeds under normal storage conditions may be associated with anti-aging capacity. The results of gene/protein interaction analyses suggest that miR164c first targets *PSK5*, and the PSK5 protein then interacts with the ubiquitin-associated gene *RPS27AA*, which simultaneously impacts the genes/proteins in the six above-mentioned functional categories. Expression levels of some of the key genes and proteins in the interaction network were verified by real-time fluorescence quantitative PCR (RT-qPCR) and multiple reaction monitoring mass spectrometry (MRM-MS), respectively. Thus, the present study provides new insights into the miRNA-mediated gene and protein interaction network that regulates seed vigor.

## Introduction

Seeds are often used to preserve plant germplasm and also as a food source. Seed vigor is a comprehensive indicator of seed quality, which not only determines the germination potential of seeds under diverse field conditions but also affects the ability of plants to resist environmental stresses and to maximize the production potential (Rajjou et al., 2012). Seed vigor is formed gradually during development and usually reaches a peak at physiological maturity. However, seed vigor gradually decreases under natural storage conditions via the natural aging process. The rate of seed aging indicates the speed of decline of seed vigor. The anti-aging capacity of seeds is directly correlated with seed vigor: the stronger the anti-aging capacity, the longer the seeds will maintain high vigor. Therefore, anti-aging capacity is essential for the longevity of seeds during storage. Since the natural aging of seeds is a relatively slow process, it is difficult to measure changes in seed vigor over a short duration of time. Therefore, artificial aging is usually used to accelerate the speed of seed vigor decline and to evaluate the storage tolerance of seeds by imitating the natural aging process (Freitas et al., 2006). To induce artificial aging, seeds are exposed to a high temperature (approximately 41°C) and high relative humidity (RH; 100%) for a designated duration. Then, a germination test is carried out to examine changes in seed vigor (Arc et al., 2011; Dantas et al., 2019). Aging affects several physiological and biochemical processes within the seed, including reactive oxygen species (ROS) metabolism, malondialdehyde (MDA) content, lipid peroxidation, membrane permeability (Mira et al., 2011; Chen et al., 2016), nucleic acid stability, and protein expression and modification (Rajjou and Debeaujon, 2008; Kranner et al., 2011). However, the molecular basis of anti-aging in seeds remains unclear.

MicroRNAs (miRNAs) are endogenous, 21–24 nt long non-coding RNAs found in plants, animals, and some microorganisms. The main role of miRNAs is to silence the target mRNAs by pairing with complementary bases, resulting in either degradation of target mRNAs or inhibition of translation (Bartel, 2009, 2018). The regulatory roles of miRNAs are evident in almost every aspect of plant physiological and metabolic processes, including growth, development, and biotic and abiotic stress responses (Khraiwesh et al., 2012; Sun, 2012). Additionally, miRNAs have been reported to affect seed vigor. In rice (*Oryza sativa* L.), the expression of miR164c is negatively correlated with seed vigor (Zhou et al., 2020). However, the mechanism underlying miR164c-mediated regulation of rice seed vigor or anti-aging capacity has not yet been determined.

With the recent progress in functional genomics research and advances in high-throughput technologies, including RNA-sequencing (RNA-seq) and tandem mass tags (TMTs), it is possible to infer transient gene expression in cells or tissues at the whole-genome level, determine the gene and protein networks that regulate a particular biological phenomenon or process, screen potential key regulatory genes, and develop new approaches for crop improvement at the molecular level (Ge et al., 2003; Kumar et al., 2016). The transcriptome and proteome refer to the entire set of RNAs and proteins, respectively, generated in cells (cell populations), tissues, organs, or organisms under specific physiological conditions (Wilkins, 2009). The genome of a wild-type (WT) organism is usually stable and consistent; however, the transcriptome and proteome can easily change during growth and development, and under different environmental stimuli or stresses. To date, there have been several reports on the transcriptomic and proteomic analyses of seed vigor. For example, it has been shown that heat shock proteins (HSPs), late embryogenesis abundant proteins (LEAs), and seed storage proteins are closely related to seed vigor (Rajjou et al., 2008; Catusse et al., 2011). Another study showed that energy metabolism-related proteins, proteolytic enzymes, endosperm proteins, and glycolytic-related enzymes show significant changes in seeds upon artificial aging treatment, suggesting that these proteins are related to seed vigor (Zhang et al., 2016). Despite the above reports, the mechanism of miRNA-mediated regulation of seed vigor remains unknown.

Rice is one of the most widely cultivated crops in the world and represents the staple food of nearly 50% of the global population, especially in Asian countries. Rice is the third highest yielding crop after corn and sugarcane (UN Food and Agriculture Organization, 2017). Given its small genome and abundant genetic resources, rice is considered a model monocot (Ishibashi et al., 2018). However, the vitality of rice seeds, especially hybrid rice seeds, decreases easily during natural storage, which has huge adverse effects on food production and economic benefits (Ishibashi et al., 2018). To date, no effective strategy has been developed to increase the longevity of rice seeds. Therefore, understanding the molecular regulatory mechanism of rice seed vitality is critical, as it would help to guide the development of genetic engineering methods to improve storage tolerance and maintain high vitality of rice seeds, which has important social and economic implications for humankind.

In the present study, high-throughput RNA-seq and high-resolution mass spectrometry using TMTs were performed to determine the transcriptome and proteome, respectively, of the seeds of the WT rice cultivar “Kasalath” and its transgenic derivatives, MIM164c (miR164c-silenced line) and OE164c (miR164c-overexpression line). Functional enrichment analyses were conducted to screen genes/proteins potentially related to the anti-aging capacity of rice seeds. To verify the accuracy of omics data, real-time fluorescence quantitative PCR (RT-qPCR) and multiple reaction monitoring mass spectrometry (MRM-MS) were used to determine the expression level of genes and proteins, respectively, in seeds before and after artificial aging. The MERLIN iterative algorithm and String database were used to predict the gene/protein interaction network that regulates miR164c-guided seed vigor. Overall, our data provide new insights into the miRNA-mediated gene and protein interaction network that regulates seed vigor.

## Materials and Methods

### Plant Materials

Rice (*O. sativa* L.) cultivar “Kasalath” (WT), miR164c-silenced line L13-1-2-1 (MIM164c; hereafter referred to as ST), and miR164c overexpression line L4-1-3-1 (OE164c; hereafter referred to as OT) were used in this study. Both transgenic lines were generated in “Kasalath” background by the Plant Development and Molecular Laboratory of Hunan Normal University, China, as described previously (Zhou et al., 2020). The ST and OT seeds used in this study were in the T_5_ generation. In order to enable an assessment of significance, quantitative analyses using omics (transcriptomics, proteomics), seed morphological phenotype and germination, and qPCR were performed with a minimum of three independent biological replicates per genotype.

### Artificial Aging Treatment and Germination Test

Healthy WT and transgenic seeds of the same size and at the same maturity level were treated with high temperature (43 ± 2°C) and RH (100%) for 8 days, and then tested for germination, as described previously (Zhou et al., 2020).

### RNA Extraction

Embryos were excised from each seed sample and ground to a fine powder in liquid nitrogen. The ground tissue was transferred to a pre-chilled 1.5-mL Eppendorf tube, and total RNA was isolated using the TRIzol^®^ Reagent (Life Technologies, Carlsbad, CA, United States), according to the manufacturer’s instructions.

### Transcriptome Sequencing

The quality of the isolated total RNA was monitored by electrophoresis using 1% agarose gels. RNA purity was checked using the NanoPhotometer^®^ spectrophotometer (IMPLEN, Westlake Village, CA, United States), and RNA concentration was measured using the Qubit^®^ RNA Assay Kit in the Qubit^®^ 2.0 Fluorometer (Life Technologies). The integrity of total RNA was assessed using the RNA Nano 6000 Assay Kit of the Bioanalyzer 2100 system (Agilent Technologies, Santa Clara, CA, United States). Sequencing libraries were prepared using NEBNext^®^ Ultra^TM^ RNA Library Prep Kit for Illumina^®^ (NEB, Ipswich, MA, United States), according to the manufacturer’s recommendations, and index codes were added to identify each sample. Briefly, mRNA was purified from 3 μg total RNA using oligo-d(T) magnetic beads. The purified mRNA was fragmented at an elevated temperature using divalent cations in the NEBNext First Strand Synthesis Reaction Buffer (5×). First-strand cDNA was synthesized using random hexamer primers and M-MuLV Reverse Transcriptase (RNase H^–^). Second-strand cDNA synthesis was subsequently performed using DNA Polymerase I and RNase H. The remaining overhangs were converted into blunt ends using exonuclease/polymerase. The cDNA fragments were 3′ adenylated and then ligated to the NEBNext Adaptor with a hairpin loop structure. To select cDNA fragments ranging in length from 150 to 200 bp, samples were purified using the AMPure XP system (Beckman Coulter, Beverly, MA, United States). Then, 3 μL USER Enzyme (NEB) was incubated with size-selected, adaptor-ligated cDNAs for 15 min at 37°C and then at 95°C for 5 min. Subsequently, PCR was performed using Phusion High-Fidelity DNA polymerase, Universal PCR primers, and Index (X) Primer. The PCR products were purified using the AMPure XP system, and library quality was assessed on the Agilent Bioanalyzer 2100 system.

Clustering of index-coded samples was performed on a cBot Cluster Generation System using TruSeq PE Cluster Kit v3-cBot-HS (Illumina, San Diego, CA, United States), according to the manufacturer’s instructions. After cluster generation, library preparations were sequenced on the Illumina Hiseq platform, and 125–150 bp paired-end reads were generated.

### RNA-seq Data Analysis

Raw sequence reads in FASTQ format were first processed using in-house Perl scripts. Reads containing adapter sequences and poly-Ns, as well as low-quality reads, were removed from the raw data to obtain clean reads. The Q20 (base call accuracy = 99%) and Q30 (base call accuracy = 99.9%) quality scores and GC contents of clean reads were calculated. All subsequent analyses were performed using high-quality clean reads.

Reference genome and gene model annotation files were downloaded from the Rice Annotation Project Database (RAP-DB) website^1^. Index of the reference genome was built using Bowtie v2.2.3, and paired-end clean reads were aligned to the reference genome using TopHat v2.0.12.

HTSeq v0.6.1 was used to count the number of reads mapped to each gene. Then, values of FPKM (fragments per kilobase of transcript sequence per million base pairs) of each gene were calculated based on the length of the gene and read counts mapped to the gene, as described previously (Trapnell et al., 2010).

Differential gene expression analysis of two treatments (aged and unaged seeds) for each of the three genotypes (WT, ST, and OT; three biological replicates per treatment) was performed using the DESeq R package (1.18.0). The resulting *P*-values were adjusted using the Benjamini–Hochberg approach for controlling the false discovery rate (FDR). Genes with an adjusted *P*-value < 0.05 were designated as differentially expressed.

The Kyoto Encyclopedia of Genes and Genomes (KEGG) database was used to identify enriched pathways. A two-tailed Fisher’s exact test was performed to test the enrichment of the differentially expressed genes (DEGs) against all identified genes using the R package clusterProfiler. The pathway with a corrected *P*-value < 0.05 was considered significant. These pathways were classified into hierarchical categories according to the KEGG website.

### Protein Extraction

Total protein was extracted from the excised embryos. Briefly, embryos were first ground in liquid nitrogen for 30 min and then sonicated three times on ice using a high-intensity ultrasonic processor (Scientz) in lysis buffer [8 M urea, 2 mM EDTA, 10 mM dithiothreitol (DTT; Sigma), and 1% Protease Inhibitor Cocktail (Sigma–Aldrich)]. The debris was removed by centrifugation at 20,000 × *g* at 4°C for 10 min. Protein was precipitated with cold 15% trichloroacetic acid (TCA) at −20°C for 4 h. The sample was centrifuged at 12,000 × *g* at 4°C for 3 min, and the supernatant was discarded. The protein pellet was washed three times with cold acetone, and then redissolved in a buffer containing 8 M urea and 100 mM tetraethylammonium bromide (TEAB; pH 8.0). Protein concentration was determined using the 2-D Quant Kit (GE Healthcare), according to the manufacturer’s instructions.

### Protein Digestion, TMT Labeling, and High-Performance Liquid Chromatography (HPLC) Fractionation

Protein digestion and TMT labeling were conducted according to the method of Wang et al. (2018). The protein sample was then fractionated by high-pH reversed-phase high-performance liquid chromatography (HPLC) using an Agilent 300 Extend C18 column [5 μm particle size, 4.6 mm internal diameter (i.d.), 250 mm length]. Briefly, peptides were first separated into 80 fractions with a gradient of 2–60% ACN in 10 mM ammonium bicarbonate (pH 9.0) over 80 min. Then, peptides were combined into 18 fractions and dried by vacuum centrifugation.

### Liquid Chromatography-Tandem Mass Spectrometry (LC-MS/MS) Analysis

Peptides were dissolved in 0.1% formic acid (FA; Fluka) and then directly loaded onto a reversed-phase pre-column (Acclaim PepMap 100, Thermo). Peptide separation was performed using a reversed-phase analytical column (Acclaim PepMap RSLC, Thermo). The gradient comprised an increase from 5 to 25% solvent B (0.1% FA in 98% ACN) in 26 min, 25 to 40% B in 8 min, 40 to 80% B in 3 min, and then holding at 80% B for the last 3 min, all at a constant flow rate of 350 nL/min on an EASY-nLC 1000 ultra performance liquid chromatography (UPLC) system.

Peptides were subjected to an NSI source, followed by MS/MS in Q Exactive^TM^ (Thermo) coupled with UPLC online. Intact peptides were detected in the orbitrap at a resolution of 70,000. Peptides were selected for MS/MS using the NCE setting of 28, and ion fragments were detected in the orbitrap at a resolution of 17,500. A data-dependent procedure that alternated between one MS scan followed by 20 MS/MS scans was applied for the top 20 precursor ions above a threshold ion count of 1E4 in the MS survey scan, with 30.0 s dynamic exclusion. The electrospray voltage applied was 2.0 kV. Automatic gain control (AGC) was used to prevent overfilling of the orbitrap, and 5E4 ions were accumulated to generate the MS/MS spectra. MS scans were performed at a mass-to-charge ratio (*m/z*) ranging from 350 to 1,800, and the fixed first mass was set at 100 *m/z*.

### Proteome Data Analysis

The resulting MS/MS data were processed using MaxQuant with an integrated Andromeda search engine (v.1.5.2.8). Tandem mass spectra were searched against the transcriptome database. Trypsin/P was specified as the cleavage enzyme, allowing up to two missed cleavages. Mass error was set to 10 ppm for precursor ions and 0.02 Da for fragment ions. Carbamidomethylation of cysteine (Cys) residues was specified as a fixed modification, and oxidation of methionine (Met) and acetylation of the protein N-terminus were specified as variable modifications. To perform protein quantification, TMT 6-plex was selected in Mascot. The FDR was adjusted to <1% at the protein, peptide, and peptide-spectrum match (PSM) level.

To ensure that the sample preparation met the standard requirements, the mass error of all identified peptides was first checked. The distribution of mass error should be approximately zero and the concentration of most of the peptides should be <10 ppm, which enable the mass accuracy of MS data to fit the requirements. Then, the length of peptides was verified; most peptides should be 8–16 amino acids (aa) in length, which is consistent with the property of tryptic peptides.

Gene Ontology (GO) annotation of the proteome was derived from the UniProt-GOA database^2^. First, the identified protein ID was converted to UniProt ID and then mapped to GO IDs. If any of the identified proteins could not be annotated using the UniProt-GOA database, the InterProScan soft was then used to annotate the GO function, based on protein sequence alignment. Then, proteins were classified into three GO categories: Biological Process, Cellular Component, and Molecular Function. In each category, the enrichment of the differentially expressed proteins (DEPs) against all identified proteins was determined using the two-tailed Fisher’s exact test. Correction for multiple hypothesis testing was carried out using the standard FDR control methods. The GO with a corrected *P*-value < 0.05 was considered significant.

The KEGG database was used to identify enriched pathways, and statistical analysis was conducted as described above for GO categories. The KEGG pathways were classified into hierarchical categories according to the KEGG website.

Functional enrichment (GO and KEGG)-based clustering of different protein groups was used to explore the potential relationships among different protein groups. All protein groups obtained after functional enrichment analysis, along with their *P*-values, were first collated and then filtered for categories enriched in at least one of the protein groups with *P-*value < 0.05. This filtered *P-*value matrix was transformed by the function x = −log_10_(*P-*value). Finally, these x values were z-transformed for each functional category. The z scores were then clustered by one-way hierarchical clustering (Euclidean distance, average linkage clustering). Cluster membership was visualized using a heat map constructed with the heatmap.2 function in the gplots R package.

MapMan was used to analyze the proteome data according to the method of Klie and Nikoloski (2012). First, the mapping file of the annotation result was obtained by submitting nucleotide sequences of the transcriptome profiling results to Mercator4 v1.0^3^. Then, DEPs together with the MapMan ontology information of corresponding genes were selected, and a two-tailed Fisher’s exact test was employed for each comparison group to test the enrichment of DEPs against all mapped proteins. The MapMan terms with a corrected *P*-value < 0.05 were considered significant.

### Correlated Proteome and Transcriptome Quantification

In proteome profiling, proteins with a quantitative ratio above 1.3 or below 0.77 (1/1.3) were deemed significantly differentially expressed. In transcriptome profiling, genes with a fold-change (FC) > 1.2 and corrected *P-*value < 0.05 were deemed significantly differentially expressed. To obtain further biological information, functional enrichment of genes/proteins in different crosstalk categories was analyzed. GO and KEGG pathway enrichment clustering analyses were performed using the same method as in protein profiling.

### Network Analysis

The modular regulatory network learning with per gene information (MERLIN) algorithm (Roy et al., 2013) was used to infer the regulatory network of the genes-of-interest in transcriptome profiling. The transcriptome data obtained in this study and those downloaded from the National Center Biotechnology Information (NCBI) were used. The data matrix comprised 164 samples from 13 experiments. First, FPKM of the genes were transformed into transcripts per million reads (TPM), and the mean expression level of each gene was calculated. Then, the expression levels of genes were zero-mean transformed. Genes were retained for the MERLIN algorithm only if (1) their expression value varied by at least ± 1 from the mean in at least five samples and (2) they were in the list of DEGs and DEPs in the transcriptome and proteome data, respectively, obtained in the present study. A total of 30 sub-sets were created from the amended data matrix, each of which contained 50% of the samples selected randomly from the complete matrix. Data from each sub-set were used to infer a MERLIN interaction. In the final MERLIN network, each edge (which indicates the relation between two genes) appeared at least 18 times in the 30 sub-sets (confidence = 60%).

To further predict interactions, the DEPs in the proteome (P), proteins corresponding to the target genes of miR164c (T), and proteins corresponding to the DEGs in the transcriptome that interact with P or T in the above MERLIN interactions, were used as the input in the String database v11.0^4^, with medium confidence (40%). Target genes of miR164c were mostly obtained via psRNATarget^5^ and from the results of degradome sequencing of WT, ST, and OT (unpublished data).

Finally, the MERLIN and String network were integrated, and the Cytoscape v3.7.1 software was used to develop an interaction network comprising miR164c target genes as well as DEGs and DEPs. All genes and proteins in the interaction network were ranked by six ranking methods using cytoHubba of the Cytoscape v3.7.1 software. The top 10 genes/proteins obtained by each ranking method were compared, and those common among all six methods were termed as hub genes/proteins (Sang et al., 2018).

### MRM-MS Analysis

To extract protein, seed embryos were ground to a fine powder in liquid nitrogen and extracted with lysis buffer [7 M urea, 2 M thiourea, 4% CHAPS, and 40 mM Tris-HCl (pH 8.5)], containing 1 mM PMSF and 1 mM EDTA (final concentration). The further procedures for protein preparation were the same as the method of Liu et al. (2018).

To digest the proteins, 100 μg of total protein was sampled and digested with Trypsin Gold (Promega, Madison, WI, United States) at a trypsin:protein mass ratio of 1:30 at 37°C for 16 h. After trypsin digestion, peptides were dried by vacuum centrifugation and reconstituted in 0.5 M TEAB.

To conduct LC-MRM-MS, samples were digested with trypsin, as described above, and spiked with 50 fmol β-galactosidase for data normalization. MRM analyses were performed on a QTRAP 5500 mass spectrometer (SCIEX, Framingham, MA, United States) equipped with an LC-20AD nano HPLC system (Shimadzu, Kyoto, Japan). The mobile phase consisted of solvent A (0.1% aqueous FA) and solvent B (98% ACN in 0.1% FA). Peptides were separated on a C18 column (0.075 × 150 mm column, 3.6 μm) at a flow rate of 300 L/min. Peptide samples were then eluted at a gradient of 5–30% solvent B for 38 min and 30–80% solvent B for 4 min, followed by a hold at 80% solvent B for 8 min. The QTRAP5500 mass spectrometer was used at an electrospray voltage of 2,400 V, nebulizer gas of 23 psi, and dwell time of 10 ms. To maximize specificity, several MRM transitions were monitored using unit resolution in both Q1 and Q3 quadrupoles.

For transition selection, a spectral library of MS/MS data was generated on TripleTOF5600 (AB SCIEX, Foster City, CA, United States) and searched using Mascot v2.3 (Matrix Science, London, United Kingdom) against the rice database (122,753 entries) downloaded from the UniProt Knowledgebase (UniProtKB). The data file was imported into the Skyline software where a library was built. To develop the MRM method, peptides were selected according to the following criteria: (1) peptide sequence was unique in the UniProtKB rice database; (2) maximum peptide *m/z* < 1,250 (limitation of the Quadrupole scan), and peptide length = 5–40 aa; (3) no Met residues in peptides; (4) carbamidomethylation of Cys residues, without variable modification in peptides; and (5) no missed cleavage by trypsin. Initially, six transitions per peptide were monitored to ensure specificity according to the following criteria: (1) fragment ions were in the form of b- or y-ions; (2) precursor ion charge was 2, 3, or 4, and the fragment ion charge was 1 or 2. The predicted retention time of targeted peptides was observed with an iRT strategy. The pooled peptides were digested as described above and subjected to preliminary selective reaction monitoring (SRM) assays to determine where the proteins were detected.

To validate the MRM method, chromatograms of all transitions generated on QTRAP5500 (SCIEX) were input into Skyline. The MRM method was considered successful for a given protein only if the protein produced at least one unique peptide with the following characteristics: (1) identified with MS/MS spectral library cut-off score > 0.95; (2) showed more than five fragment ions with the same elution profile and the same ratio as the spectral library; and (3) showed an accurate retention time (less than ± 2 min deviation from the predicted retention time).

To analyze the LC-MRM-MS data, the Skyline software was used to integrate the raw file generated by QTRAP 5500. An iRT strategy was used to predict the retention time of a given peptide against a spectral library. All transitions for each peptide were used for quantitation, unless interference was observed from the matrix. A spike of β-galactosidase was used for label-free data normalization. MSstats with the linear mixed-effects model was used for data analysis. Three technical replicates were performed for each sample. The *P-*value was adjusted to control the FDR at a cut-off of 0.05. All proteins with a *P*-value < 0.05 and FC > 1.5 were considered statistically significant.

### RT-qPCR Assay

The RT-qPCR assay was conducted as described previously (Zhou et al., 2020). Forward primers used for miRNA genes, and primers used for other genes, are listed in Supplementary Table S4. The expected size of the amplified fragments varied from 80 to 200 bp. Three technical replicates were performed for each sample. Statistical analysis was performed using Piko Real Software 2.0, and a paired *t*-test was performed to compare expression levels.

## Results

### Anti-aging Capacity and miR164c Expression Level Differ Among WT, ST, and OT Seeds

**FIGURE 1:**
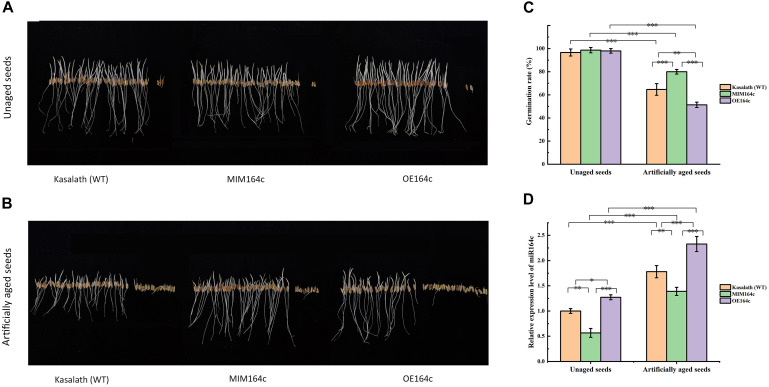
Analysis of seed morphological phenotypes and miR164c expression levels in the seeds of the wild-type rice cultivar “Kasalath” (WT), miRNA164c-silenced line (MIM164c; ST), and miR164c overexpression line (OE164c; OT) before and after artificial aging. **(A,B)** Morphological phenotypes of germinating seeds. **(C)** Seed germination rates. **(D)** miR164c expression levels. Photographs shown in **A** and **B** were taken on the third day of germination. Data represent mean ± SD (*n* = 3). Significant differences in seed germination rates and miR164c expression levels among the different rice genotypes were determined using Tukey’s test (^∗^*P* < 0.05, ^∗∗^*P* < 0.01, ^∗∗∗^*P* < 0.001). In **D**, the expression level of miR164c in unaged WT seeds was defined as 1.

The germination rates of unaged “Kasalath” (WT), MIM164c (ST), and OE164c (OT) seeds were 96.7, 98, and 98.7%, respectively, thus showing no significant differences (Figures 1A,C). However, after 8 day of artificial aging, the germination rates of all three genotypes decreased significantly to 64.7, 80, and 51.3%, respectively (Figures 1B,C). Thus, aged seeds of the three genotypes showed significantly different germination rates (*P* < 0.01), along with morphological differences (Figures 1B,C). Additionally, the expression level of miR164c in the aged seeds of all three genotypes was significantly higher than that in the corresponding unaged seeds (*P* < 0.001). On the other hand, compared with WT seeds, the expression level of miR164c was significantly lower in ST seeds (*P* < 0.01) and higher in OT seeds (*P* < 0.05 for unaged seeds; *P* < 0.001 for artificially aged seeds), irrespective of the aging treatment. These results are consistent with those of a previous report (Zhou et al., 2020).

### Differences in the Transcriptome and Proteome of WT, ST, and OT Seeds

The transcriptome is a collection of all RNA molecules in a cell or group of cells, which reflects the expression status of the entire genome. The transcriptome data and quality of WT, ST, and OT seeds are shown in Supplementary Table 1. The HTSeq software and “union” model were used to analyze the gene expression level. A total of 91,992 genes were obtained (Supplementary Table 2), of which approximately 75,000 genes showed FPKM values ranging from 0 to 1; approximately 7,000 genes showed FPKM values in the range of 3–15; between 1,580 and 1,864 genes showed FPKM values > 60.

**FIGURE 2:**
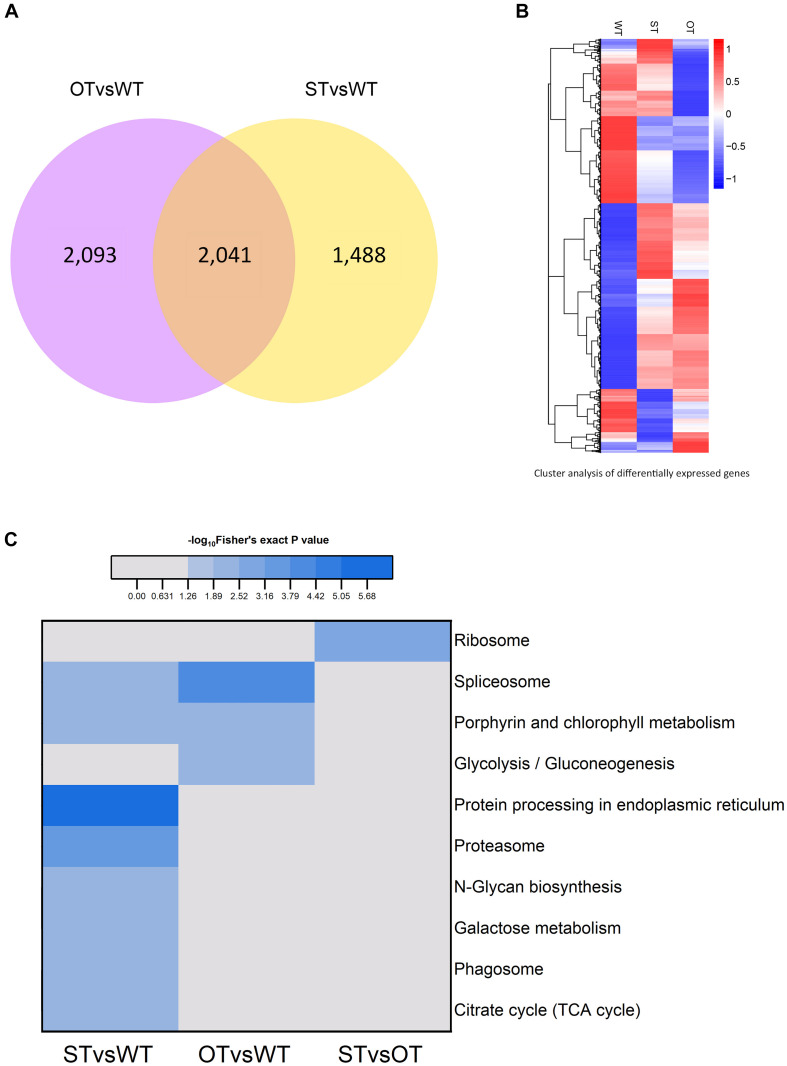
Analysis of the transcriptome of ST and OT seeds relative to that of WT seeds (three biological replicates per genotype). **(A)** Venn diagram. **(B)** Cluster analysis. In **B**, the greater the intensity of the red color, the higher the gene expression; and the greater the intensity of the blue color, the lower the gene expression. **(C)** Heat map showing the results of KEGG pathway enrichment analysis of genes differentially expressed between WT, MIM164c (ST), and OE164c (OT) seeds.

The number of genes differentially expressed between WT and ST seeds (WTvsST), and between WT and OT seeds (WTvsOT), was 3,529 and 4,134, respectively, of which 2,041 genes were common to both groups (Figure 2A). Cluster analysis showed that the expression patterns of the DEGs differed greatly among the three genotypes (Figure 2B), suggesting that the differential expression of miR164c among these genotypes likely has an important impact on these genes.

The results of KEGG pathway enrichment analysis of the DEGs are shown in Figure 2C. In total, 10 different pathways were significantly enriched (*P* < 0.05): eight in STvsWT, three in OTvsWT, and one in STvsOT. Among these pathways, “spliceosome” and “porphyrin and chlorophyll metabolism” were common between the STvsWT and OTvsWT groups. Notably, the “protein processing in endoplasmic reticulum (ER)” KEGG pathway was the most significantly enriched and the only enriched pathway among DEGs identified in the STvsWT comparison. The ER is the main site of intracellular protein processing, protein folding, and transport of newly synthesized proteins to the Golgi apparatus. Studies show that HSPs in the ER can help to maintain standard protein folding and regulate plant defense response against abiotic stresses (Lindquist and Craig, 1988; Xu et al., 2005). Moreover, HSPs in the ER can enhance the anti-aging capacity of seeds (Rajjou and Debeaujon, 2008). In this study, the seeds of MIM164c possessed the highest anti-aging capacity. Therefore, genes in this ER-related pathway may play key roles in the regulation of the anti-aging capacity of seeds.

**FIGURE 3:**
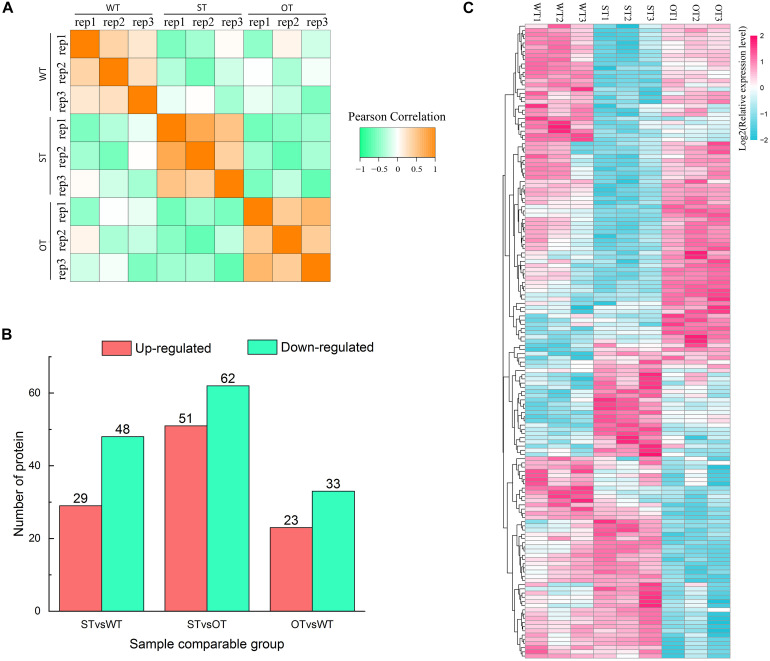
Proteome analysis of WT, ST, and OT seeds (three biological replicates per genotype). **(A)** Reproducibility of the proteome data. **(B)** Number of DEPs. **(C)** Cluster analysis of DEPs.

Protein is the primary bearer of life activities. To further investigate the molecular basis of the differences in the anti-aging capacity of WT, ST, and OT seeds, proteomic analyses were performed using the TMT method. A total of 4,066 proteins were identified, of which 3,604 proteins had quantitative information. The repeatability analysis of each sample showed that the protein quantification results were reliable (Figure 3A). In this study, proteins with FC > 1.3 or < 0.77, and *P*-value < 0.05, were considered as differentially expressed. Based on these criteria, the number of DEPs was 77 (29 up-regulated and 48 down-regulated) in STvsWT, 113 (51 up-regulated and 62 down-regulated) in STvsOT, and 56 (23 up-regulated and 33 down-regulated) in OTvsWT (Figure 3B). Cluster analysis of DEPs showed that only a few proteins showed similar levels between ST and OT seeds (Figure 3C), indicating that the differential expression of miR164c in two transgenic lines may contribute to differences in protein expression.

**FIGURE 4:**
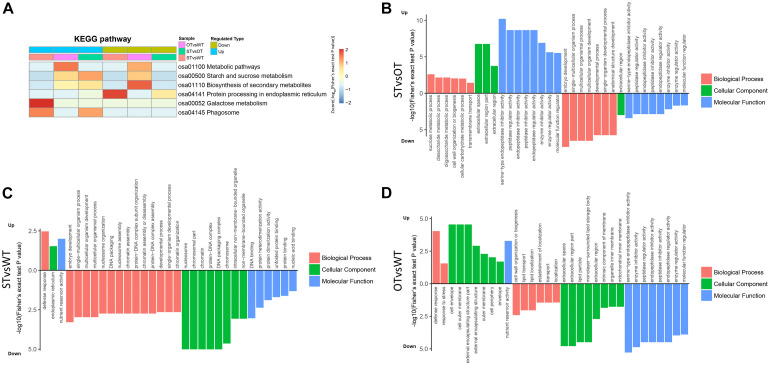
Heat map showing the results of KEGG and GO enrichment analyses of DEPs in WT, ST, and OT seeds. **(A)** KEGG enrichment analysis. The greater the intensity of the red color, the greater the degree of enrichment. GO enrichment analyses of DEGs identified in STvsOT **(B)**, STvsWT **(C)**, and OTvsWT **(D)** comparisons.

KEGG enrichment analysis showed that the DEPs identified above were significantly (*P* < 0.05) enriched in six pathways, namely, “metabolic pathways” (osa01100), “starch and sucrose metabolism” (osa00500), “biosynthesis of secondary metabolites” (osa01110), “protein processing in ER” (osa04141), “galactose metabolism” (osa00052), and “phagosome” (osa04145) (Figure 4A). Among these pathways, the enrichment of three pathways was consistent with the results of the transcriptome KEGG enrichment analysis (Figure 2C): “protein processing in ER” significantly (*P* < 0.05) enriched in the down-regulated proteins in both STvsWT and STvsOT groups; “phagosome,” a pathway related to cell phagocytosis, significantly (*P* < 0.05) enriched in the up-regulated proteins in both STvsWT and STvsOT groups; and “galactose metabolism,” an energy metabolism-related pathway, significantly (*P* < 0.05) enriched in the up-regulated proteins in the STvsWT group.

In addition to KEGG analysis, GO enrichment analysis of the DEPs in the STvsOT, STvsWT, and OTvsWT groups was also performed (Figures 4B–D). The GO terms were categorized under three broad categories: Biological Process, Cellular Component, and Molecular Function. In the STvsOT, STvsWT, and OTvsWT groups, according to −log_10_(Fisher’s exact test *P*-value), DEPs were significantly enriched in 33 GO terms (including 13 terms in the Biological Process category, four terms in Cellular Component, and 16 terms in Molecular Function); 31 GO terms (15 terms in Biological Process, nine terms in Cellular Component, and seven terms in Molecular Function); and 32 GO terms (eight terms in Biological Process, 15 terms in Cellular Component, and nine terms in Molecular Function), respectively. In each GO category, the terms enriched in the up-regulated and down-regulated proteins showed significant differences among the STvsOT, STvsWT, and OTvsWT groups. For example, compared with STvsWT and OTvsWT, most terms were enriched in the Molecular Function category in STvsOT. It is worth noting that each enriched term in this category in the STvsOT group contained both up-regulated and down-regulated proteins, and the number of up-regulated proteins was greater than that of down-regulated proteins (Supplementary Figure 1). All of these GO terms, except “molecular function regulator,” were mainly related to peptidase activity with high significance. On the other hand, the same down-regulated peptidase-related GO terms were also enriched in the Molecular Function category in OTvsWT, but none of these terms were enriched in STvsWT. These results suggest that the differential expression of miR164c alters the physiological and metabolic status of seeds of the three rice lines at the protein level.

**FIGURE 5:**
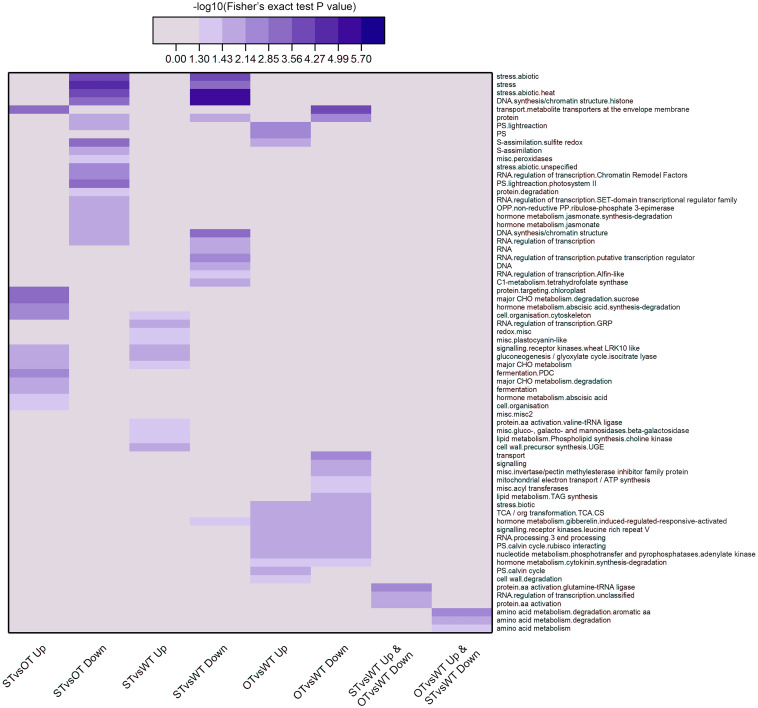
Clustering analysis of the results of MapMan pathway enrichment of genes corresponding to the DEPs identified in the WT, ST, and OT seeds. The darker the color, the more significant the degree of enrichment. The DEPs were selected based only on fold-change (FC > 1.3 or < 1/1.3), and not on the basis of the *P*-value.

Next, we used MapMan, a plant gene annotation database, to annotate genes corresponding to the DEPs identified in this study. MapMan pathway enrichment was performed at Levels 1–3 for all DEPs with FC ≥ 1.3 (Figure 5). To obtain as much enrichment information as possible, the *P*-value of the DEPs was not considered during MapMan enrichment analysis. The results showed that the DEPs were significantly enriched (Fisher’s exact test *P-*value < 0.05) in a total of 68 pathways, among which, according to −log_10_(Fisher’s exact test *P*-value), the top 10 pathways in descending order were as follows: “stress.abiotic.heat,” “DNA.synthesis/chromatin structure.histone,” “stress,” “transport.metabolite transporters at the envelope membrane,” “stress.abiotic,” “major CHO metabolism.degradation.sucrose,” “DNA.synthesis/chromatin structure,” “protein.targeting.chloroplast,” “S-assimilation.sulfite redox,” “PS.lightreaction.photosystem II.” More than half of these pathways were potentially related to the anti-aging capacity of seeds. For example, “stress” and “stress.abiotic.heat” pathways were significantly enriched among the down-regulated proteins in both STvsOT and STvsWT groups; the “transport.metabolite transporters at the envelope membrane” pathway was significantly enriched among the up-regulated and down-regulated proteins in STvsOT and OTvsWT, respectively; “CHO metabolism.degradation.sucrose,” a major pathway related to energy metabolism, was significantly enriched among the up-regulated proteins in STvsOT; and two pathways potentially related to gene expression regulation, namely, “DNA.synthesis/chromatin structure.histone” and “DNA.synthesis/chromatin structure,” were significantly enriched among the down-regulated proteins in both STvsOT and STvsWT groups.

### Correlation Analysis Between the Seed Transcriptome and Proteome

**FIGURE 6:**
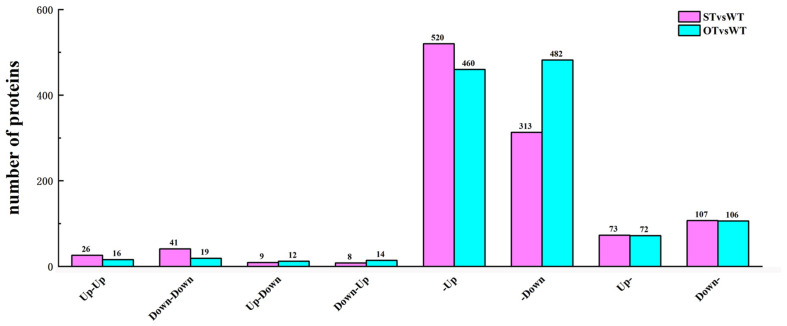
Number of DEGs/DEPs in each of the eight transcriptome–proteome correlation categories. Up-Up: up-regulated in both proteome and transcriptome; Down-Down: down-regulated in both proteome and transcriptome; Up-Down: up-regulated in proteome and down-regulated in transcriptome; Down-Up: down-regulated in proteome and up-regulated in transcriptome; -Up: unchanged in proteome and up-regulated in transcriptome; -Down: unchanged in proteome and down-regulated in transcriptome; Up-: up-regulated in proteome and unchanged in transcriptome; Down-: down-regulated in proteome and unchanged in transcriptome.

In the transcriptome, up-regulated genes with FC > 1.2, down-regulated genes with FC < 1/1.2, and all genes with the corrected *P*-value < 0.05 were designated as DEGs. In the proteome, up-regulated proteins with FC > 1.3 and down-regulated proteins with FC < 1/1.3 were designated as DEPs. Based on the correlation between the DEGs and DEPs in STvsWT and OTvsWT groups, the genes/proteins were divided into eight categories: (1) up-regulated in both the transcriptome and proteome; (2) down-regulated in both the transcriptome and proteome; (3) down-regulated in the transcriptome but up-regulated in the proteome; (4) up-regulated in the transcriptome but down-regulated in the proteome; (5) up-regulated in the transcriptome but unchanged in the proteome; (6) down-regulated in the transcriptome but unchanged in the proteome; (7) unchanged in the transcriptome but up-regulated in the proteome; and (8) unchanged in the transcriptome but down-regulated in the proteome. A total of 1,097 genes/proteins were identified in these eight categories in the STvsWT group (Figure 6), of which 92.3% belonged to four categories (520 genes/proteins in category #5, 313 in #6, 73 in #7, and 107 in #8). In OTvsWT, a total of 1,181 genes/proteins were in the eight categories (Figure 6), and 94.8% of these belonged to categories #5 (460), #6 (482), #7 (72), and #8 (106). These results indicate that the transcription level of an overwhelming majority of genes is not consistent with their translation level, implying that gene transcription and translation might be relatively independent; this was supported by previous studies (Gygi et al., 1999; Maier et al., 2009; He et al., 2016).

**FIGURE 7:**
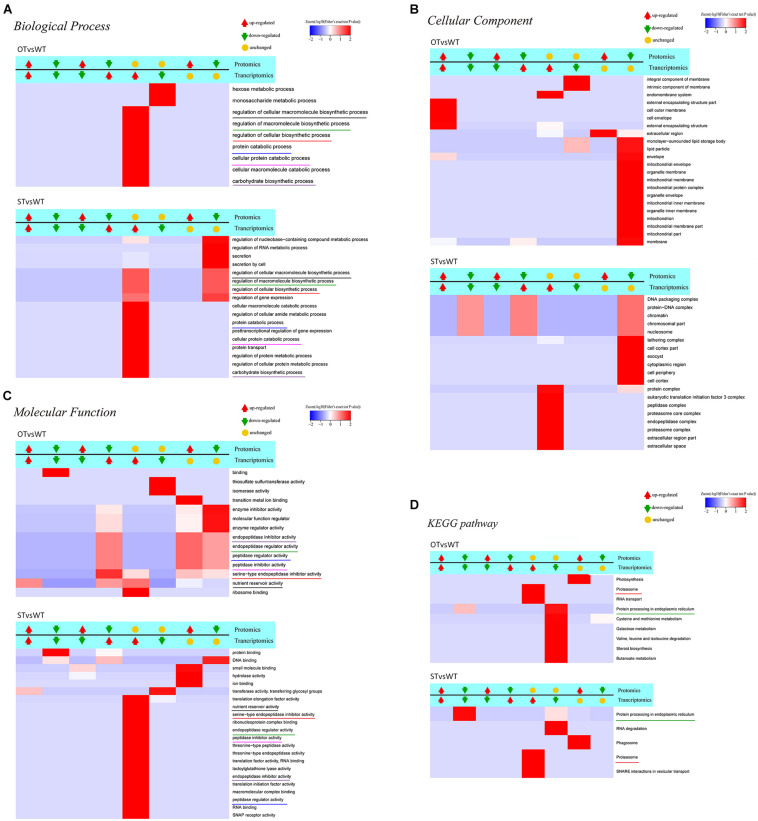
Clustering analysis of the results of functional enrichment of DEGs/DEPs identified by transcriptome–proteome correlation analysis. The common GO terms and KEGG pathways in the STvsWT and OTvsWT groups are underlined by the same color. The darker the red color, the more significant the degree of enrichment. **(A–C)** The GO categories Biological Process, Cellular Component, and Molecular Function, respectively. **(D)** represents the KEGG pathway enrichment of DEGs.

We further performed GO function and KEGG pathway enrichment analyses on DEGs/DEPs identified in the abovementioned eight categories and then clustered the enriched terms or pathways (Figure 7). Since the Biological Process category contained too many levels, only Level 6 and Level 7 GO terms were considered in this category; however, all levels were considered in the Molecular Function and Cellular Component categories. In Biological Process, 17 and nine GO terms were enriched in STvsWT and OTvsWT, respectively, of which six terms are common to both groups (Figure 7A). In Cellular Component, 19 and 22 GO terms were enriched in STvsWT and OTvsWT, respectively, with no common GO terms (Figure 7B). In Molecular Function, 14 and 22 GO terms were enriched in OTvsWT and STvsWT, respectively, of which six GO terms were common to both groups (Figure 7C). The results of KEGG pathway enrichment analysis showed that five and nine pathways were enriched in STvsWT and OTvsWT, respectively, of which two pathways were common to both groups; however, one of the two common pathways varied greatly in enrichment between STvsWT and OTvsWT (Figure 7D).

To identify genes/proteins relevant to the anti-aging capacity of seeds, based on the transcriptome–proteome correlation analysis, six significantly enriched common GO terms, but with significantly different enrichment between STvsWT and OTvsWT groups, were noteworthy in the Molecular Function category (Figure 7C): “endopeptidase inhibitor activity,” “endopeptidase regulator activity,” “peptidase regulator activity,” “peptidase inhibitor activity,” “serine-type endopeptidase inhibitor activity,” and “nutrient reservoir activity.” All of these GO terms, except “nutrient reservoir activity,” were peptidase-related, and the DEGs and DEPs enriched in these five GO terms belonged to category #5 in STvsWT; however, in OTvsWT, the expression correlation between these DEGs and DEPs was diverse. In addition, among these five GO terms, “serine-type endopeptidase inhibitor activity” was the most significantly enriched, with genes/proteins up-regulated in the transcriptome and down-regulated in the proteome of OTvsWT (Figure 7C), which was consistent with the results of GO enrichment analysis of DEPs in STvsOT and OTvsWT (Figures 4B,D). Genes in the “serine-type endopeptidase inhibitor activity” GO term were up-regulated in the transcriptome in both STvsWT and OTvsWT groups, indicating that ST and OT seeds showed higher expression level of serine endopeptidase inhibitor-related genes than WT seeds. However, compared with the WT, the level of proteins corresponding to these genes showed significant differences between the two transgenic lines, i.e., unchanged in ST seeds and down-regulated in OT seeds. We speculate that the difference in the expression level of serine-type endopeptidase inhibitory-related proteins between ST and OT seeds was mainly caused by different regulatory mechanisms guided by the differential expression of miR164c between the two transgenic lines.

The significantly enriched GO terms that were unique and accounted for a larger proportion of all GO terms in each of the two groups, STvsWT and OTvsWT, were mainly found in the Cellular Component category (Figure 7B). Among these GO terms, seven mitochondrial-related terms were notably enriched and accounted for more than one-third of all 22 significantly enriched GO terms in OTvsWT, including “mitochondrial envelope,” “mitochondrial membrane,” “mitochondrial protein complex,” “mitochondrial inner membrane,” “mitochondrion,” “mitochondrial membrane part,” and “mitochondrial part.” The expression of all genes in these GO terms was unchanged in the transcriptome, while all proteins in these GO terms were down-regulated in the proteome. However, these genes/proteins were not significantly enriched in the STvsWT group. This result is consistent with the GO enrichment result of the DEPs in the mitochondria-related terms, and the MapMan enrichment result of the TCA-related pathway in OTvsWT (Figures 4D, 5). The mitochondria-related proteins were significantly down-regulated in OTvsWT, indicating that the energy metabolism of OT seeds may be abnormal to some degree.

In the KEGG pathway enrichment results (Figure 7D), two significant enriched pathways, “proteasome” and “protein processing in ER,” were common to both STvsWT and OTvsWT groups. However, the “proteasome” pathway showed very similar correlation categories between STvsWT and OTvsWT, while the “protein processing in ER” pathway showed highly distinct categories between STvsWT and OTvsWT (#6 in OTvsWT and #2 in STvsWT). The significant down-regulation of ER-related proteins in the STvsWT group was consistent with the results of KEGG enrichment analysis of the DEGs in the transcriptome (Figure 2C) and the DEPs in the proteome (Figure 4A). We speculate that the differential expression of ER-related proteins between ST and OT seeds was mainly caused by different regulatory mechanisms guided by the differential expression of miR164c between the two transgenic genotypes.

### Key Genes/Proteins Associated With the Anti-aging Capacity of Rice Seeds

**FIGURE 8:**
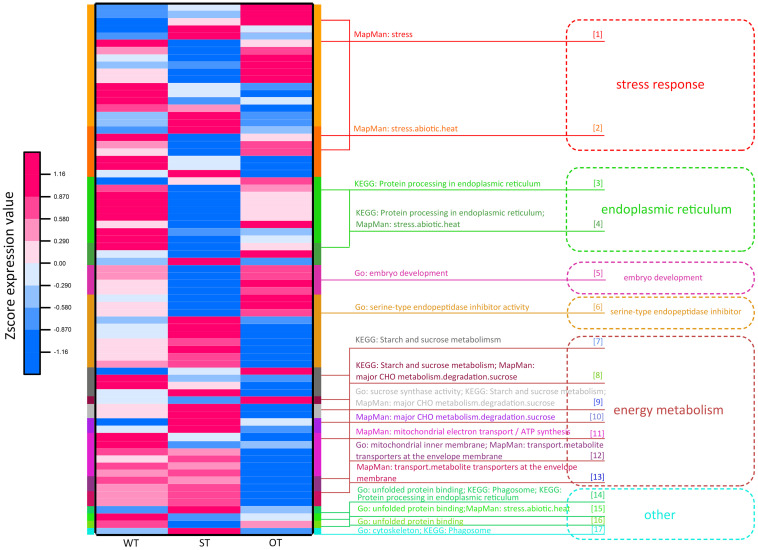
Functional cluster heat map showing the expression levels of DEPs in WT, ST, and OT seeds.

Through the transcriptome and proteome functional enrichment analyses and transcriptome–proteome correlation analysis of WT, ST, and OT seeds, some key KEGG/MapMan pathways and GO terms that may be related to the anti-aging capacity of rice seeds were obtained. These included GO terms such as “serine endopeptidase,” “embryo development,” “energy metabolism,” and “protein folding”; KEGG pathways such as “ER protein processing,” “energy metabolism,” “phagosomes,” and “other”; and MapMan pathways such as “stress and energy metabolism.” All DEPs could be divided into six categories, based on their function: “stress response,” “ER,” “embryo development,” “serine-type endopeptidase inhibitor,” “energy metabolism,” and “other.” The “other” category included difficult-to-classify proteins such as the unfolded protein-related protein and phagosome-related protein (Supplementary Table 3). Cluster analysis was performed based on the expression levels of these proteins (Figure 8). The results showed that the expression patterns of energy metabolism-related proteins were similar between ST and WT seeds, and the expression levels of these proteins were higher in ST and WT seeds than in OT seeds. Expression patterns of most proteins related to “stress response,” “ER protein processing,” “embryo development,” and “other” functions were similar between OT and WT seeds, and their expression levels were higher in OT and WT seeds than in ST seeds. Additionally, most serine endopeptidase inhibitor- and sucrose synthase-related proteins in the “energy metabolism” category were expressed in seeds in the following order: ST > WT > OT; this is consistent with the anti-aging capacity of rice seeds. However, the expression level of proteins related to embryo development followed the order OT > WT > ST, which was exactly opposite to the anti-aging capacity of seeds. We further compared the relative expression levels of the six key types of DEGs/DEPs between seeds of the three rice genotypes using the transcriptome and proteome data (Supplementary Figure 2). The results showed that, irrespective of the transcriptome or proteome, the most consistent differences in expression between the three seed types were in serine endopeptidase inhibitor- and energy metabolism-related genes/proteins. Thus, we speculate that differences in the expression levels of serine endopeptidase inhibitor- and energy metabolism-related proteins may be associated with the anti-aging capacity of rice seeds.

Protein involved in stress response, ER protein processing, protein folding, and embryo development (LEA proteins in this study; Supplementary Table 3) are collectively referred to as stress-related proteins (Lindquist and Craig, 1988; Cuming, 1999; Xu et al., 2005; Kalemba and Pukacka, 2008; Rajjou and Debeaujon, 2008). Most of these proteins were expressed to higher levels in OT and WT seeds than in ST seeds (Figure 8). This implies that under the unaged condition, high expression of stress-related proteins in seeds may be unfavorable for maintenance of high vigor.

### Interaction Between miR164c Target Genes and DEGs/DEPs

How miR164c interacts with genes/proteins in the six major functional categories to regulate seed vigor was not clear. Here, we investigated the interaction between the miR164c target genes and DEGs/DEPs identified in this study using the MERLIN algorithm, a highly accurate and iterative algorithm based on gene expression.

**FIGURE 9:**
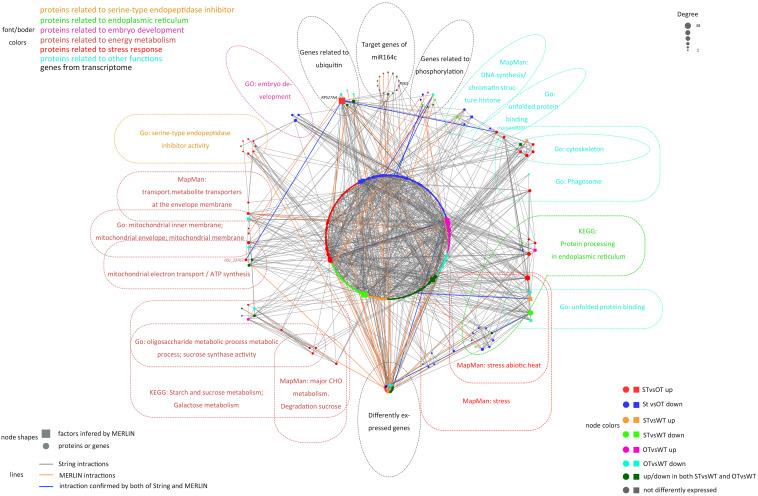
Interaction between miR164c target genes and DEGs/DEPs identified in the transcriptome and proteome of WT, ST, and OT seeds. Colors of different boxes and the text within each box represent the major functional categories of genes or proteins. Colored nodes represent different types of regulation. Node shapes represent different types of genes or proteins. The node size represents the degree of nodes; the bigger the node size, the higher the degree. DEPs were identified based on the value of FC alone (>1.3 or <1/1.3). DEGs were identified using thresholds for FC (>1.2 or <1/1.2) as well as the *P*-value (<0.05).

The MERLIN algorithm mainly performs regulatory predictions between the genes to be analyzed (Target genes) and the regulatory factors (Factor) that affect gene expression (such as transcription factors and kinases), based on massive gene expression data (Roy et al., 2013). In this study, transcriptomes of 164 rice samples (including the transcriptome obtained in this study plus transcriptomes downloaded from NCBI) were used as the data matrix. The DEGs in the transcriptome and genes corresponding to the DEPs in the proteome in the present study, as well as the target genes of miR164c, were considered as Target genes. Genes corresponding to the DEPs in the proteome, genes related to gene expression regulation (such as those encoding transcription factors and phosphorylation, acetylation, methylation, and ubiquitination enzymes) in the transcriptome, as well as target genes of miR164c, were considered as Factors. Altogether, 6,178 Target genes and 719 Factors were used as the input for the MERLIN algorithm. The results revealed 811 interactions among 331 Factors and 769 Target genes. Additionally, the String database predicted 1,049 interactions among 385 proteins. In the integrated MERLIN and String network, 14 miR164c target genes and more than 400 DEGs/DEPs were identified in the seeds of the three rice genotypes (Figure 9 and Supplementary Table S5).

The network included 76.7% (indicated in bold in Supplementary Table 3) of all the proteins in the six categories obtained by the functional enrichment analysis of the transcriptome and proteome. Some of the proteins in each functional category were closely interrelated, including a number of common proteins in the “stress response,” “ER,” and “other” (unfolded protein-related) categories; this is consistent with the result of protein expression cluster analysis (Figure 8). The network also shows that the serine endopeptidase inhibitors were closely associated with embryo development- and energy metabolism-related proteins.

**FIGURE 10:**
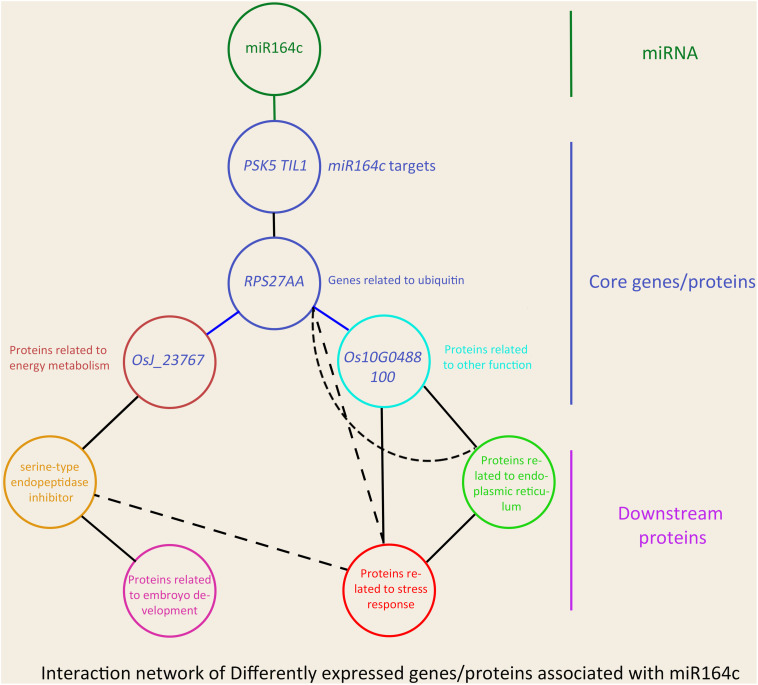
Schematic showing the effect of miR164c on rice seed vigor via *RPS27AA* in the functional gene/protein interaction network. Solid straight lines represent strong interaction, and dashed lines represent weak interaction. The color of the outer frame of each node is the same as that of the functional proteins in Figure 9. Blue lines represent the interactions confirmed both by the MERLIN algorithm and String database.

In the network, five hub genes including *RPS27AA*, *RPL5A*, *RACK1B*, *OS10G0569200*, and *OS08G0559200* were selected using six ranking methods in cytoHubba. Among these genes, *RPS27AA* was ranked as the first gene in the results of three out of six methods (Table 1). *RPS27AA* was also the hub node in the network confirmed by String and MERLIN algorithm together (Figure 9). *RPS27AA* was identified as a DEG in the STvsOT group. *RPS27AA* is related to ubiquitination, and interacts with two miR164c target genes (*OsTIL1* and *Os06g0622500*), and simultaneously connects DEPs related to energy metabolism and protein folding in “other” functional categories, which then interact with proteins in the other four functional categories (“stress response,” “ER,” “serine endopeptidase inhibitor,” and “embryo development”). Figure 10 is a simplified diagram showing the *RPS27AA*-mediated interactions between miR164c target genes and genes/proteins in the six functional categories. It is puzzling that neither *TIL1* nor *Os06g0622500*, which interacts with *RPS27AA* in the network, was differentially expressed; however, another target gene of miR164c, *PSK5*, which does not interact directly with *RPS27AA* in the network, showed differential expression in the transcriptome of seeds of the three rice genotypes. Additionally, *TIL1* showed interaction with *PSK5*, which positively regulates the anti-aging capacity of rice seeds (Zhou et al., 2020). This raises the questions of how *PSK5* interacts with *RPS27AA* to regulate seed vigor, and whether *TIL1* is involved in this regulation. The network analysis reveals clear relationships among the six types of functions, indicating that the proteins may play potential roles in the anti-aging ability of the rice seeds. Given their pivotal role in connecting miR164c with genes/proteins in the six functional categories, we designated *PSK5*, *RPS27AA*, *OsJ_23767*, and *Os10G0488100* as the core genes (Figure 10).

**TABLE 1:** Ranking of hub genes in the gene/protein interaction network using cytoHubba.

**Rank**	**Ranking methods in cytoHubba^†^**					
	**MCC**	**MNC**	**Degree**	**EPC**	**Closeness**	**Radiality**
1	***RACK1B***	***RPS27AA***	***RPS27AA***	*OS06G0608300*	***RPS27AA***	*OS06G0608300*
2	***RPL5A***	*OS06G0608300*	*OS06G0608300*	*OsJ_13577*	*OS06G0608300*	***RPS27AA***
3	***OS08G0559200***	*OsJ_13577*	*DJC43*	***RPS27AA***	*OsJ_13577*	***RACK1B***
4	*OS07G0224000*	*OS04G0107900*	*OsJ_13577*	***RPL5A***	*OS04G0107900*	***OS08G0559200***
5	*OsJ_33893*	***RPL5A***	*OS04G0107900*	***OS10G0569200***	***RACK1B***	*DJC43*
6	***RPS27AA***	*DJC43*	***RACK1B***	*OsJ_25910*	*DJC43*	***RPL5A***
7	***OS10G0569200***	***RACK1B***	***OS08G0559200***	***RACK1B***	***RPL5A***	*OsJ_13577*
8	*OS07G0124500*	***OS10G0569200***	***RPL5A***	*OS07G0224000*	***OS08G0559200***	*OS04G0107900*
9	*OsJ_32984*	***OS08G0559200***	***OS10G0569200***	***OS08G0559200***	***OS10G0569200***	*OsJ_25910*
10	*OsJ_25910*	*OsJ_33893*	*OS07G0124500*	*OS04G0107900*	*OsJ_25910*	***OS10G0569200***

*^†^Genes indicated in bold represent hub genes identified within the top 10 list by each of the six ranking methods in cytoHubba. MCC, maximal clique centrality; MNC, maximum neighborhood component; Degree, node connect degree; EPC, edge percolated component; Closeness, closeness centrality; Radiality, reachability to other nodes.*

### Verification of RNA-seq Data

**FIGURE 11:**
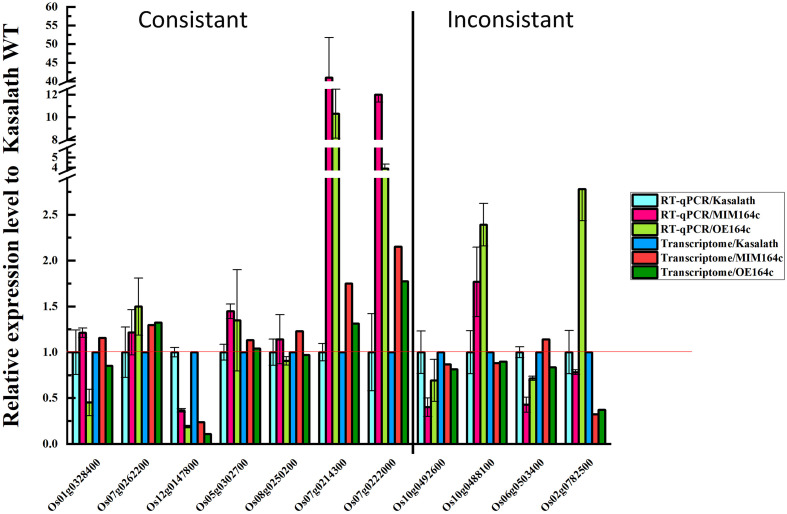
Validation of the RNA-seq data of 11 DEGs by RT-qPCR. Data represent mean ± SD (*n* = 3).

To verify the reliability of RNA-seq data, the expression of 11 DEGs with different functions was analyzed by RT-qPCR using the same batch of seeds as was used for RNA-seq. Of these 11 genes, 10 were included in the gene/protein interaction network and belonged to the six functional categories identified above (Figure 9). The RT-qPCR results of seven genes were consistent with the RNA-seq data, indicating that our transcriptome data are reliable (Figure 11). The RT-qPCR results of the other four genes were inconsistent with the RNA-seq data, possibly because of the difference between the sensitivity of the two methods (Zhao et al., 2012; Ke et al., 2014).

**FIGURE 12:**
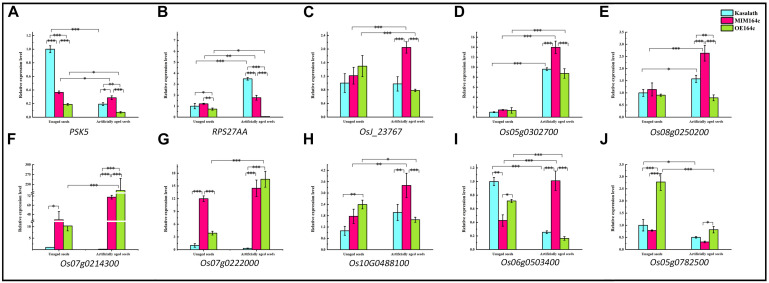
RT-qPCR assay of the expression level of 10 genes in WT, ST, and OT seeds before and after artificial aging. **(A–C,H)** Core genes in the interaction network in Figure 9. **(C–E)** Genes related to energy metabolism. **(F,G)** Genes corresponding to serine-type endopeptidase inhibitors. **(H–J)** Stress-related genes. Data represent mean ± SD (*n* = 3). Significant differences in gene expression levels among the three rice genotypes were determined by Tukey’s test (^∗^*P* < 0.05, ^∗∗^*P* < 0.01, ^∗∗∗^*P* < 0.001). The expression level of each gene in unaged WT seeds was defined as 1.

Expression levels of the above mentioned 10 genes were significantly affected by the artificial aging treatment (Figure 12), further implying that these genes may be related to the anti-aging capacity of rice seeds.

*PSK5* is one of the target genes of miR164c. RT-qPCR assay of unaged seeds showed that the expression level of *PSK5* was highest in WT seeds and lowest in OT seeds, indicating that *PSK5* expression was inhibited by genetic transformation, regardless of whether the transformation was for miR164c silencing or miR164c overexpression, but the inhibitory effect of miR164c overexpression was more severe than that of miR164c silencing. Following the artificial aging treatment, the expression level of *PSK5* decreased in the seeds of all genotypes; however, *PSK5* expression was highest in ST seeds and lowest in OT seeds (Figure 12A). These results were consistent with the differences in the seed anti-aging capacities of the three genotypes and opposite to the expression levels of miR164c in these genotypes (Figure 1D). On the other hand, the expression level of *RPS27AA* in unaged ST and WT seeds was significantly higher than that in unaged OT seeds, and artificial aging exacerbated this difference (Figure 12B). The expression level of *RPS27AA*, *OsJ_23767*, and *Os10G0488100* significantly increased in ST seeds and significantly decreased in OT seeds after artificial aging. Although up to 14 target genes of miR164c were involved in the interaction network, only *PSK5* showed differential expression among the three rice lines. Whether the other miR164c target genes are involved in the regulation of seed vigor remains unclear.

Expression analysis of three energy metabolism-related genes (*OsJ_23767*, *Os05G0302700*, and *Os08t0250200*; Figures 12C–E), an unfolded protein-related gene (*Os10G0488100*; Figure 12H), and a stress response-related gene (*Os06G0503400*; Figure 12I) by RT-qPCR showed that all five genes were significantly up-regulated in artificially aged ST seeds relative to unaged ST seeds. Moreover, among artificially aged seeds, the expression level of these five genes was higher in ST seeds and lower in OT seeds relative to the WT. Differences in the expression levels of these five genes among the artificially aged seeds of the three rice genotypes were highly consistent with those of *PSK5*. These results suggest that miR164c regulates seed vigor via its target gene *PSK5*, which interacts with *RPS27AA* to affect the downstream genes.

Expression levels of two serine endopeptidase inhibitor-related genes, *Os07g0214300* and *Os07g0222006*, increased in ST and OT seeds after artificial aging; however, both these genes were expressed to a lower level in WT seeds, regardless of the aging treatment (Figures 12F,G). This indicates that T-DNA transformation may increase the expression of these genes, and the stress of aging may further enhance this increase, especially in OT seeds. Compared with WT seeds (aged or unaged), the expression level of the ER-related gene *Os02G0782500* (Figure 12J) was lower in ST seeds and higher in OT seeds, indicating that the expression of *Os02G0782500* was inhibited by the silencing of miR164c and promoted by the overexpression of miR164c, but the stress of aging significantly suppressed its expression in seeds of all genotypes. This result is consistent with the expression level of the corresponding stress response- and ER-related proteins shown in Figure 8.

### Verification of TMT Data

**FIGURE 13:**
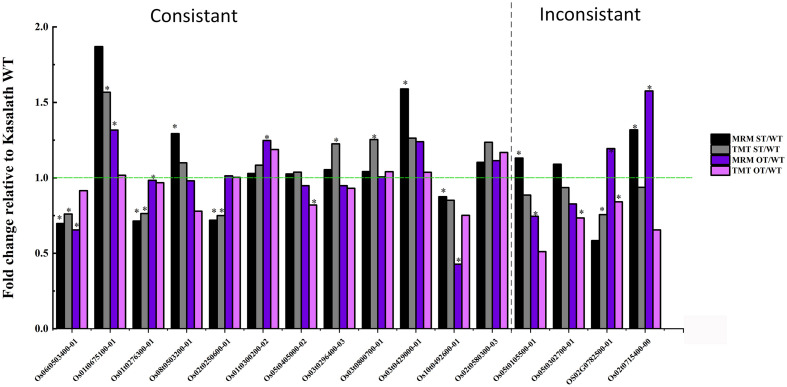
Comparison of the quantitative data of 16 rice seed proteins between MRM-MS and TMT assays. Data represent the mean of three technical replicates. Asterisk (^∗^) indicates the significant difference (a linear mixed-effects model was used for MRM data and a paired *t*-test for TMT data) in the protein expression level between transgenic and WT seeds (*P* < 0.05). The expression level of each protein in WT seeds was defined as 1.

To verify the TMT data, the expression of 60 randomly selected 1.2-fold changed DEPs was verified by MRM-MS. Of these 60 proteins, 16 were successfully quantified by MRM-MS (Figure 13), and the results of 12 of the 16 proteins (75%) were consistent with the proteomics data, indicating that our proteomics data are reliable. The reason that most of the DEPs could not be quantitatively verified by MRM-MS may be the strict analysis conditions and workflow of MRM. This also shows that MRM-MS analysis has certain limitations for the verification of proteomics results.

**FIGURE 14:**
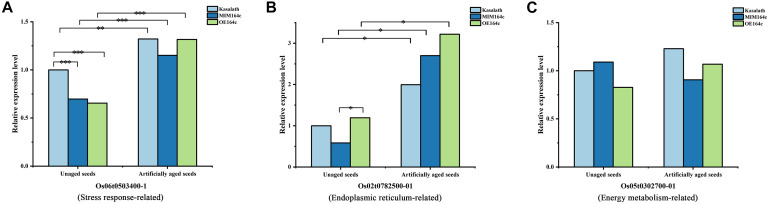
Quantitative results of three key proteins in aged and unaged WT, ST, and OT seeds by MRM-MS. Data represent the mean of three technical replicates. A linear mixed-effects model was used to determine significant differences in protein expression levels among the three rice genotypes (^∗^*P* < 0.05, ^∗∗^*P* < 0.01, ^∗∗∗^*P* < 0.001). The expression level of each protein in unaged WT seeds was defined as 1.

Of the 16 proteins successfully quantified by MRM-MS, three proteins (Os06t0503400-01, Os05t0302700-01, and Os02t0782500-01) were members of the gene/protein interaction network (Figure 9). The expression of genes corresponding to these three proteins was also verified by RT-qPCR. Compared with unaged seeds, the expression level of *Os05G0302700* and *Os02G0782500* was up-regulated and down-regulated, respectively, in the aged seeds of all three rice genotypes, whereas *Os06G0503400* was up-regulated in aged ST seeds and down-regulated in aged WT and OT seeds (Figure 12). Bioinformatics analyses showed that Os06t0503400-01, Os05t0302700-01, and Os02t0782500-01 are stress response-, energy metabolism-, and ER-related proteins, respectively. The results of MRM-MS assay showed that expression levels of both Os06t0503400-01 and Os02t0782500-01 in all three types of rice seeds increased significantly after aging, indicating that the two proteins may participate in the regulation of the anti-aging capacity of rice seeds. However, the expression level of Os05t0302700-01 did not change significantly with aging (Figure 14C), suggesting that this protein may have little to do with the regulation of seed anti-aging capacity. The difference between the results of RT-qPCR and MRM-MS for the three genes/proteins further illustrates the different regulatory mechanisms of gene transcription and translation.

## Discussion

The decline in the seed vigor of rice cultivars, especially hybrid cultivars, during storage is a serious problem (Gao et al., 2016). Investigation of the molecular basis of seed vigor or anti-aging capacity in rice will facilitate the development of new technologies that can prolong the longevity of rice seeds. The regulatory roles of miRNAs in plants are evident in almost all aspects of physiology, including the response to various stresses such as nutrient deficiency, drought, and cold (Khraiwesh et al., 2012; Sun, 2012). Identification of the regulatory mechanisms of miRNAs will help to improve the yield and stress resistance of crops (Djami-Tchatchou et al., 2017).

Studies show that miRNAs affect seed vigor in rice, and the expression level of miR164c is negatively correlated with rice seed vigor (Zhou et al., 2020). Our results confirmed the differences in the anti-aging capacities and miR164c expression levels among WT, ST, and OT seeds (Figure 1). Moreover, using transcriptomic and proteomic approaches, we elucidate the miR164c-guided gene/protein interaction network involved in the regulation of rice seed vigor (Figures 9, 13).

### Six Types of Proteins May Be Involved in the Regulation of the Anti-aging Capacity of Rice Seeds

Many studies have shown that ER-related proteins, LEA proteins, and protein folding-related proteins are involved in stress responses and play important roles in maintaining seed vigor during storage (Lindquist and Craig, 1988; Cuming, 1999; Xu et al., 2005; Kalemba and Pukacka, 2008; Rajjou and Debeaujon, 2008; Kaur et al., 2015). The results of transcriptome and proteome analyses of WT, ST, and OT seeds indicated that six types of DEPs may be involved in the regulation of anti-aging capacity, including stress-related proteins, ER-related, embryo development-related, serine-type endopeptidase inhibitor, energy metabolism-related, and “other” proteins (such as unfolded protein-related protein and phagosome-related protein). The results of clustering analysis showed that these proteins possessed two characteristics. First, most proteins associated with stress response, such as ER-related protein, embryo development-related protein, and “other” proteins (unfolded protein-related), showed exceptionally low expression levels in ST seeds compared with WT and OT seeds. Second, the expression level of most proteins related to serine endopeptidase inhibitors and energy metabolism in ST seeds and WT seeds was higher than that in OT seeds (Figure 8). These results suggest that compared with WT seeds, active energy metabolism, inactive serine endopeptidase, and low expression of stress-related proteins in non-aged seeds contributed to the higher anti-aging capacity of ST seeds, while abnormal energy metabolism, active serine endopeptidase, and high expression of stress-related proteins in unaged seeds accelerated the aging of OT seeds.

Studies have shown that proteins related to stress response, ER, and embryo development (LEA proteins), as well as unfolded proteins (in the “other” category in this study), are all associated with stress response to a certain extent (Lindquist and Craig, 1988; Cuming, 1999; Xu et al., 2005; Kalemba and Pukacka, 2008; Rajjou and Debeaujon, 2008). In the present study, the expression level of the stress response-related protein (Os06t0503400-01) and ER-related protein (Os02t0782500-01) increased significantly in seeds of all three rice genotypes after artificial aging (Figures 14A,B). Molecular chaperones such as proteins involved in protein folding or misfolding and ER-associated degradation (ERAD) are related to unfolded proteins and are reported to play important roles in the response to ER stress, which occurs when the level of unfolded or misfolded proteins increases because of environmental stress-induced increase in the redox level, calcium concentration, or ATP level in the ER lumen (Hetz and Papa, 2018). An accumulation of unfolded proteins in the ER and mitochondria triggers stress responses or unfolded protein responses (UPRs), potentially triggering cell death and many aging-related neurodegenerative diseases (Schröder and Kaufman, 2005; Xu et al., 2005; Bernales et al., 2012). In the present study, gene/protein interaction analyses showed that three types of proteins (stress response-related, ER-related, and unfolded protein binding-related proteins in the “other” category) closely interacted with each other (Figure 9). The proteomics data showed that the expression level of stress-related proteins in unaged ST seeds was much lower than that in WT and OT seeds (Figure 8). Under aging stress, the expression of stress-related genes, such as *Os06t0503400*, increased in ST seeds but decreased in WT and OT seeds, which possess lower anti-aging capacity than ST seeds (Figure 12I), implying that the early high-level expression of some of the stress-related proteins in unaged seeds may be detrimental to their anti-aging capacity. Nonetheless, further research is needed to understand the intrinsic anti-aging mechanism of rice seeds.

The primary function of peptidase is to hydrolyze proteins involved in various biochemical processes. Abnormal, damaged, and short-lived regulatory proteins need to be hydrolyzed to prevent negative effects. The degradation of storage proteins in seeds provides nutrients for seed germination (Fontanini and Jones, 2002). Studies show that serine endopeptidase proteins [such as root-starvation-induced protease (RSIP)] are involved in the stress response (James et al., 1996; Park and Kwak, 2008). Furthermore, serine endopeptidases also act as *de novo* proteases and are responsible for the initial degradation of the seed storage proteins (Qi et al., 1992; Hosokawa et al., 1999; Müntz et al., 2001). In this study, serine endopeptidase inhibitors and energy metabolism-related proteins showed close association with the gene/protein network (Figures 9, 10), and the expression pattern of these two types of proteins measured by the TMT method showed the strongest correlation with the RNA-seq results (Supplementary Figure 2). Additionally, the expression levels of most energy metabolism-related proteins (12 out of 17) in ST and WT seeds were higher than those in OT seeds (Figure 8); however, the serine endopeptidase activity was highest in OT seeds (based on the lowest expression of seven out of 10 serine endopeptidase inhibitors) (Figure 8). These results suggest that OT seeds potentially have an abnormal energy metabolism and higher serine endopeptidase activity, unlike ST and WT seeds.

### Gene/Protein Network of miR164c Regulates the Anti-aging Capacity of Rice Seed

Network analyses of all kinds of omics data have been widely used to explain various molecular mechanisms in plants, animals, and microbes (Rual et al., 2005; Rolland et al., 2014; Marx et al., 2016). The String database is commonly used to predict the protein–protein interaction network (Szklarczyk et al., 2019). On the other hand, the MERLIN algorithm is used to predict gene regulatory networks, based on gene expression data (Siahpirani and Roy, 2017). However, data processing using the MERLIN algorithm involves complex steps and is time-consuming, and is therefore seldom used in omics studies (Marx et al., 2016). Moreover, most of the previous omics studies on seed vigor focused mainly on the identification of specific genes or proteins, and rarely focused on gene/protein network analysis (Daniel, 2017; Li et al., 2018; Aragão et al., 2019; Wei et al., 2020). In this study, an interaction network that describes the association between miR164c target genes and DEGs/DEPs was predicted by an integrative analysis using the MERLIN algorithm and String database (Figure 9). The results showed that one of the miR164c target genes, *PSK5*, potentially interacts with a ubiquitin-related hub gene, *RPS27AA*, which then interacts with genes in six major functional categories. RT-qPCR analyses verified the expression levels of 10 closely interacting genes in the network, including four core genes (*RPS27AA*, *PSK5*, *OsJ_23767*, and *Os10G0488100*) and six functional genes (*Os08g0250200*, *Os07g0214300*, *Os07g0222000*, *Os06g0503400*, *Os03g0296400*, *Os05g0302700*), all of which were significantly affected by the artificial aging treatment (Figure 12). Expression levels of some of the proteins corresponding to these genes were also determined by the MRM-MS assay (Figure 14). The relationship between the expression of key genes in this network and the anti-aging capacity of seeds was verified.

Overall, our results suggest that miR164c first regulates the expression level of the target gene *PSK5* and then affects the expression of the core gene *RPS27AA*, which further interacts with functional genes such as those related to “energy metabolism,” “endopeptidase inhibitor,” “embryo development,” “stress response,” “ER,” and “other,” leading to differences in seed anti-aging capacity among the three rice genotypes (Figure 10A). Thus, our results provide new insights into the role of miRNAs in the regulation of seed vigor.

## Data Availability Statement

The raw RNA-seq data have been deposited in the European Nucleotide Archive (ENA) at EMBL-EBI under accession number PRJEB40895 (https://www.ebi.ac.uk/ena/browser/view/PRJEB40895). The mass spectrometry proteomics data have been deposited to the ProteomeXchange Consortium via the PRIDE partner repository with the dataset identifier PXD022093.

## Author Contributions

XJ conceived and designed the study. KH, SZ, and KS performed the experiments. YZ and FW generated the transgenic rice lines, MIM164c and OE164c. KH analyzed the data and wrote the manuscript. All authors discussed the results and approved the final manuscript.

## Conflict of Interest

The authors declare that the research was conducted in the absence of any commercial or financial relationships that could be construed as a potential conflict of interest.

## Footnotes

1 ftp://ftp.ensemblgenomes.org/pub/plants/release-40/gff3/oryza_sativa

2 http://www.ebi.ac.uk/GOA/

3 https://plabipd.de/portal/mercator4

4 https://string-db.org/

5 http://plantgrn.noble.org/psRNATarget/

## Supplementary Material

Supplementary Figure 1 DEPs in STvsOT showing significant enrichment in GO terms belonging to the Molecular Function category.

Supplementary Figure 2 Comparison of the relative expression levels of key DEGs and corresponding proteins in the WT, ST, and OT seeds.

Supplementary Table 1 Summary of the seed transcriptome data of three rice genotypes (three biological replicates per genotype).

Supplementary Table 2 Distribution of gene transcript levels in various rice seed samples.

Supplementary Table 3 List of DEPs potentially involved in the regulation of the anti-aging capacity of rice seeds.

Supplementary Table 4 List of primers for RT-qPCR of miR164c and some genes showing differential expression between WT, ST, and OT seeds.

Supplementary Table 5 Data on the interaction between nodes (i.e., genes/proteins) in the integrated network of the MERLIN algorithm and String database.
