# Supplementary figures and images for: Elucidation of the miR164c-Guided Gene/Protein Interaction Network Controlling Seed Vigor in Rice
Source: Front Plant Sci. 2020 Nov 12;11:589005. doi: 10.3389/fpls.2020.589005 (PMC7688992; doi:10.3389/fpls.2020.589005)

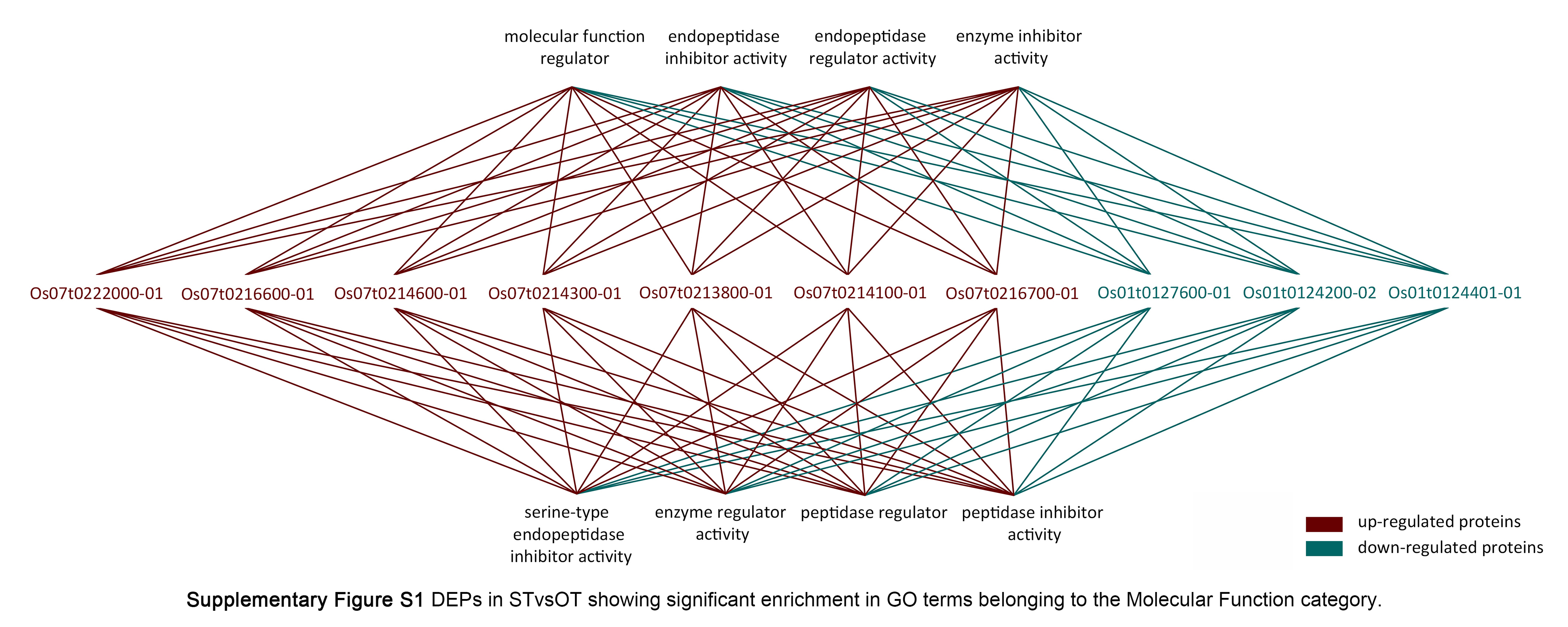

Supplement: Supplementary Figure 1 — DEPs in STvsOT showing significant enrichment in GO terms belonging to the Molecular Function category. [file Image_1.JPEG]

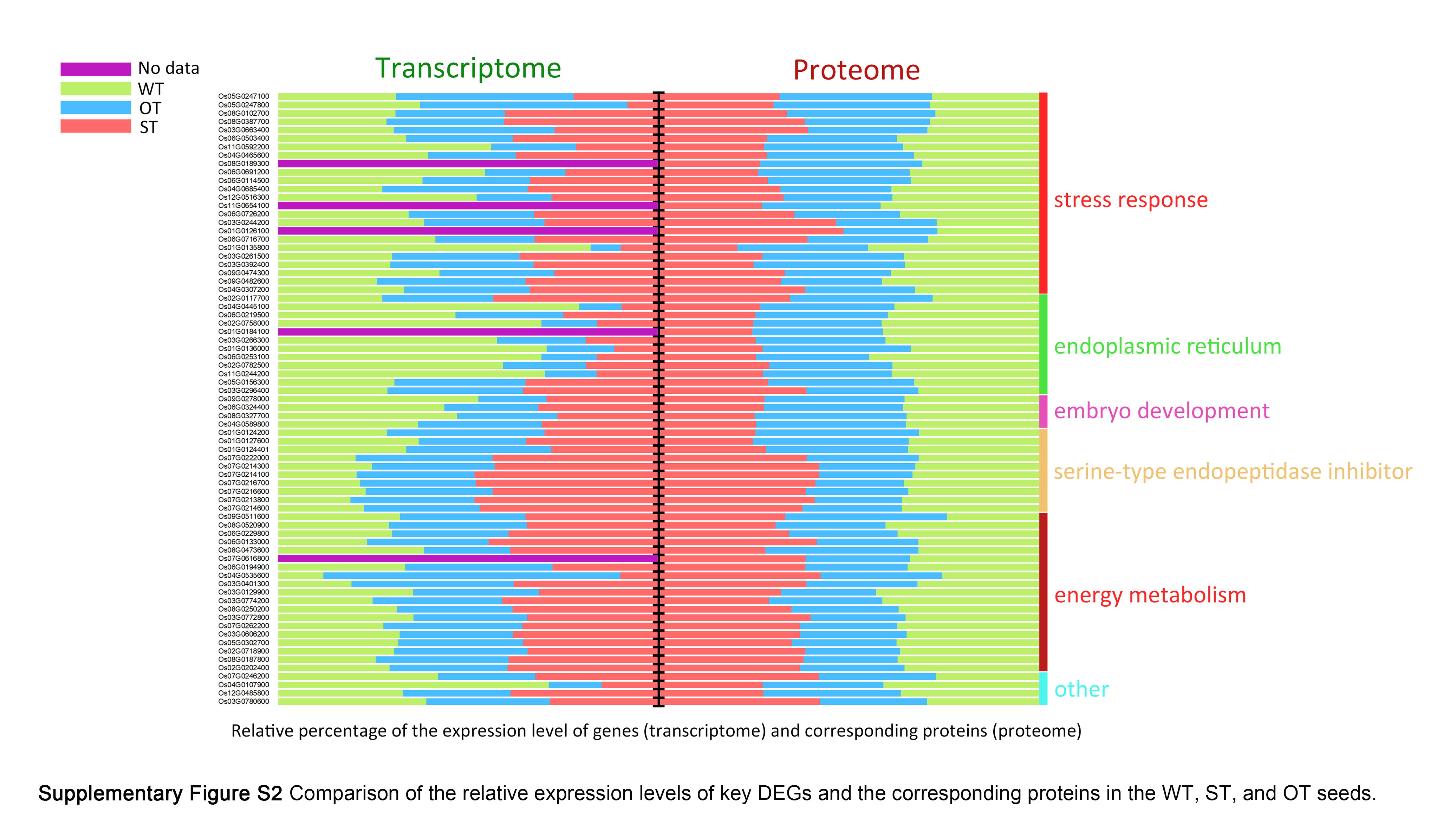

Supplement: Supplementary Figure 2 — Comparison of the relative expression levels of key DEGs and corresponding proteins in the WT, ST, and OT seeds. [file Image_2.JPEG]
