# Supplementary material for: Elucidation of the miR164c-Guided Gene/Protein Interaction Network Controlling Seed Vigor in Rice
Source: Front Plant Sci. 2020 Nov 12;11:589005. doi: 10.3389/fpls.2020.589005 (PMC7688992; doi:10.3389/fpls.2020.589005)
Supplement: Supplementary Table 1 — Summary of the seed transcriptome data of three rice genotypes (three biological replicates per genotype). [file Table_1.DOCX]

**Supplementary Table S1.** Summary of the seed transcriptome data of three rice genotypes (three biological replicates per genotype)

| **Genotype^†^** | **No. of raw reads** | **No. of clean reads** | **No. of clean bases (G)** | **Error (%)** | **Q20 (%)** | **Q30 (%)** | **GC content (%)** |
| --- | --- | --- | --- | --- | --- | --- | --- |
| WT1 | 57,771,350 | 55,506,638 | 8.33 | 0.02 | 97.22 | 93.02 | 57.35 |
| WT2 | 48,865,602 | 47,158,540 | 7.07 | 0.02 | 97.15 | 92.74 | 57.64 |
| WT3 | 58,752,920 | 56,605,158 | 8.49 | 0.02 | 97.31 | 93.10 | 57.96 |
| ST1 | 61,112,536 | 58,707,250 | 8.81 | 0.02 | 97.27 | 93.14 | 56.44 |
| ST2 | 53,218,670 | 51,375,386 | 7.71 | 0.02 | 97.18 | 92.76 | 56.89 |
| ST3 | 51,833,980 | 49,838,962 | 7.48 | 0.02 | 97.06 | 92.56 | 57.42 |
| OT1 | 48,748,660 | 47,047,284 | 7.06 | 0.02 | 96.99 | 92.46 | 56.76 |
| OT2 | 50,526,390 | 48,771,782 | 7.32 | 0.02 | 97.08 | 92.64 | 56.84 |
| OT3 | 43,867,858 | 42,290,986 | 6.34 | 0.02 | 97.22 | 92.96 | 57.02 |

^†^WT, ‘Kasalath’; ST, MIM164c; OT, OE164c. Numbers 1, 2, and 3 represent different replicates of the same sample.

**Supplementary Table S2.** Distribution of gene transcript levels in various rice seed samples

| **FPKM interval^†^** | **WT1^*^** | **WT2^*^** | **WT3^*^** | **ST1^^^** | **ST2^^^** | **ST3^^^** | **OT1^#^** | **OT2^#^** | **OT3^#^** |
| --- | --- | --- | --- | --- | --- | --- | --- | --- | --- |
| 0–1 | 75,095 (81.63%) | 74,891 (81.41%) | 75,023 (81.55%) | 74,454 (80.94%) | 74,619 (81.11%) | 74,537 (81.03%) | 74,382 (80.86%) | 74,366 (80.84%) | 74,193 (80.65%) |
| 1–3 | 4,689 (5.10%) | 4,656 (5.06%) | 4,648 (5.05%) | 4,642 (5.05%) | 4,565 (4.96%) | 4,663 (5.07%) | 4,873 (5.30%) | 4,737 (5.15%) | 4,786 (5.20%) |
| 3–15 | 7,012 (7.62%) | 7,157 (7.78%) | 7,114 (7.73%) | 6,980 (7.59%) | 6,986 (7.59%) | 7,074 (7.69%) | 7,147 (7.77%) | 7,182 (7.81%) | 7,240 (7.87%) |
| 15–60 | 3,616 3.93%) | 3,682 (4.00%) | 3,616 (3.93%) | 4,052 (4.40%) | 4,008 (4.36%) | 3,965 (4.31%) | 3,904 (4.24%) | 3,982 (4.33%) | 4,026 (4.38%) |
| >60 | 1,580 (1.72%) | 1,606 (1.75%) | 1,591 (1.73%) | 1,864 (2.03%) | 1,814 (1.97%) | 1,753 (1.91%) | 1,686 (1.83%) | 1,725 (1.88%) | 1,747 (1.90%) |
| Total | 91,992 | 91,992 | 91,992 | 91,992 | 91,992 | 91,992 | 91,992 | 91,992 | 91,992 |

^†^FPKM refers to the expected number of fragments per kilobase of transcript sequence per million reads sequenced. **^*^**WT1, WT2 and WT3 refer to three biological replicates of WT; **^^^**ST1, ST2 and ST3 refer to three biological replicates of ST; **^#^**OT1, OT2 and OT3 refer to three biological replicates of OT.

**Supplementary Table S3.** List of DEPs potentially involved in the regulation of the anti-aging capacity of rice seeds

| **Functional categories** | **Protein accession** | **Protein description** | **MW (kDa)** | **ST/WT ratio** | **OT/WT ratio** |
| --- | --- | --- | --- | --- | --- |
| stress response | Os01t0126100-01 | Cupredoxin domain containing protein | 64.049 | 1.823 | 0.926 |
|  | Os03t0244200-01 | Similar to thaumatin-like protein 1 | 28.464 | 1.738 | 0.982 |
|  | **Os03t0663400-02** | **Similar to Thaumatin-like protein** | **22.766** | **1.339** | **1.072** |
|  | **Os04t0465600-01** | **Bet v I allergen family protein** | **17.266** | **0.866** | **1.175** |
|  | Os04t0685400-02 | Harpin-induced 1 domain containing protein | 24.532 | 0.825 | 0.752 |
|  | Os05t0247800-01 | Glycoside hydrolase, family 18 protein | 32.436 | 1.053 | 1.433 |
|  | Os06t0114500-02 | Similar to ATOZI1 protein (Stress-induced protein OZI1) (AT0ZI1 protein) | 8.786 | 0.852 | 1.118 |
|  | **Os06t0503400-01** | **Reticulon family protein** | **28.59** | **0.760** | **0.915** |
|  | **Os06t0691200-01** | **Similar to Thaumatin-like protein precursor** | **25.634** | **0.770** | **1.174** |
|  | **Os06t0726200-02** | **Similar to Chitinase 1** | **33.764** | **0.976** | **0.763** |
|  | Os08t0102700-05 | Harpin-induced 1 domain containing protein | 25.209 | 1.236 | 1.431 |
|  | **Os08t0189300-01** | **Germin-like protein 8-4, Disease resistance** | **24.574** | **0.866** | **1.392** |
|  | Os05t0247100-02 | Similar to Chitinase (EC 3.2.1.14) III C00481-rice (EC 3.2.1.14) | 32.549 | 1.129 | 1.421 |
|  | Os08t0387700-03 | Hypothetical conserved gene | 73.172 | 1.342 | 1.144 |
|  | **Os11t0592200-01** | **Similar to Chitin-binding allergen Bra r 2 (Fragments)** | **16.041** | **0.777** | **1.023** |
|  | Os11t0654100-01 | NB-ARC domain containing protein | 105.29 | 0.652 | 0.749 |
|  | Os12t0516300-03 | Hypothetical conserved gene | 14.055 | 0.853 | 0.743 |
|  | **Os01t0135800-01** | **Similar to Cytosolic class I small heat shock protein 3B (Fragment)** | **16.649** | **0.460** | **0.765** |
|  | **Os03t0261500-02** | **Similar to DnaJ subfamily C member 8** | **29.237** | **0.767** | **1.050** |
|  | Os03t0392400-01 | Heat shock protein DnaJ, N-terminal domain containing protein | 42.385 | 0.708 | 1.125 |
|  | **Os04t0307200-01** | **Similar to Cysteine string protein (CCCS1)** | **76.249** | **1.181** | **0.885** |
|  | **Os06t0716700-02** | **Similar to Heat shock protein 90** | **93.044** | **1.342** | **1.090** |
|  | **Os09t0474300-01** | **Similar to Heat shock protein precursor** | **74.805** | **0.852** | **0.715** |
|  | **Os09t0482600-01** | **Similar to Heat shock protein 81-3** | **80.183** | **0.779** | **0.640** |
| endoplasmic reticulum | **Os03t0296400-03** | **Similar to Eukaryotic translation initiation factor 2 subunit 1 (Eukaryotic translation initiation factor 2 alpha subunit) (eIF-2-alpha) (EIF- 2alpha) (EIF-2A) (Fragment)** | **38.234** | **1.226** | **0.930** |
|  | **Os05t0156300-03** | **Similar to Protein disulfide isomerase** | **39.912** | **0.879** | **1.173** |
|  | **Os11t0244200-01** | **Similar to Pisum sativum 17.9 kDa heat shock protein (hsp17.9) (Fragment)** | **21.87** | **0.708** | **0.875** |
|  | **Os01t0136000-00** | **Similar to Cytosolic class I small heat shock protein HSP17.5** | **16.904** | **0.813** | **1.155** |
|  | **Os01t0184100-01** | **Similar to 17.5 kDa class II heat shock protein** | **18.031** | **0.600** | **0.839** |
|  | **Os02t0117700-01** | **UDPase** | **51.612** | **1.234** | **1.336** |
|  | **Os02t0758000-01** | **Similar to Low molecular weight heat shock protein precursor (Mitochondrial small heat shock protein 22)** | **24.097** | **0.602** | **0.816** |
|  | **Os02t0782500-01** | **Similar to Small heat stress protein class CIII** | **18.603** | **0.756** | **0.841** |
|  | **Os03t0266300-03** | **Class I low molecular weight heat shock protein 17.9** | **17.91** | **0.633** | **0.847** |
|  | **Os04t0445100-01** | **Similar to 22.7 kDa class IV heat shock protein precursor** | **23.228** | **0.703** | **0.927** |
|  | **Os06t0219500-00** | **Similar to 26.2 kDa heat shock protein, mitochondrial** | **33.533** | **0.641** | **0.876** |
|  | **Os06t0253100-01** | **Heat shock protein Hsp20 domain containing protein** | **16.023** | **0.572** | **0.665** |
| embryo development | **Os04t0589800-01** | **Late embryogenesis abundant (LEA) group 1 family protein** | **12.564** | **0.733** | **1.125** |
|  | **Os06t0324400-01** | **Late embryogenesis abundant (LEA) group 1 family protein** | **8.7166** | **0.773** | **1.023** |
|  | **Os08t0327700-01** | **Late embryogenesis abundant (LEA) group 1 family protein** | **15.163** | **0.726** | **1.147** |
|  | Os09t0278000-00 | Hypothetical conserved gene | 17.334 | 0.786 | 1.043 |
| serine-type endopeptidase inhibitor | Os01t0124200-02 | Similar to Bowman–Birk trypsin inhibitor | 27.735 | 0.806 | 1.367 |
|  | **Os01t0124401-01** | **Similar to Bowman–Birk type bran trypsin inhibitor** | **27.79** | **0.820** | **1.085** |
|  | **Os01t0127600-01** | **Similar to Bowman–Birk type proteinase inhibitor D-II precursor (IV)** | **19.334** | **0.723** | **1.192** |
|  | **Os07t0213800-01** | **Similar to Allergenic protein** | **17.27** | **1.139** | **0.637** |
|  | **Os07t0214100-01** | **Seed allergenic protein RA17 precursor** | **17.568** | **1.271** | **0.739** |
|  | **Os07t0214300-01** | **Seed allergenic protein RAG2 precursor** | **17.864** | **1.297** | **0.773** |
|  | **Os07t0214600-01** | **Similar to Seed allergenic protein RA17 precursor** | **16.982** | **1.048** | **0.723** |
|  | **Os07t0216600-01** | **Bifunctional trypsin/alpha-amylase inhibitor domain containing protein** | **16.406** | **1.128** | **0.785** |
|  | Os07t0216700-01 | Bifunctional trypsin/alpha-amylase inhibitor domain containing protein | 16.477 | 1.158 | 0.654 |
|  | **Os07t0222000-01** | **Similar to Alpha-amylase/trypsin inhibitor (RBI) (RATI)** | **15.767** | **1.233** | **0.934** |
| energy metabolism | **Os02t0718900-02** | **ADP,ATP carrier protein, mitochondrial precursor (ADP/ATP translocase) (Adenine nucleotide translocator) (ANT)** | **41.51** | **1.054** | **0.681** |
|  | **Os05t0302700-01** | **Similar to ATP/ADP carrier protein** | **40.977** | **0.935** | **0.734** |
|  | **Os07t0616800-01** | **Sucrose synthase 3 (EC 2.4.1.13) (Sucrose-UDP glucosyltransferase 3)** | **93.111** | **1.142** | **0.810** |
|  | **Os06t0194900-03** | **Sucrose synthase 2 (EC 2.4.1.13)** | **92.128** | **1.117** | **0.782** |
|  | Os06t0133000-02 | Granule-bound starch synthase 1, Starch biosynthesis | 66.475 | 1.311 | 0.842 |
|  | **Os06t0229800-01** | **Similar to Starch synthase IIA** | **88.374** | **0.923** | **0.768** |
|  | **Os08t0520900-00** | **Similar to Isoamylase (Fragment)** | **81.618** | **0.762** | **0.716** |
|  | **Os09t0511600-04** | **Glycoside hydrolase, family 1 protein** | **58.388** | **1.377** | **1.756** |
|  | **Os08t0473600-01** | **Alpha-amylase isozyme 3E precursor (EC 3.2.1.1) (1,4-alpha-D-glucan glucanohydrolase)** | **48.707** | **0.884** | **1.266** |
|  | **Os03t0401300-01** | **Sucrose synthase 2 (EC 2.4.1.13) (Sucrose-UDP glucosyltransferase 2)** | **92.907** | **1.213** | **0.910** |
|  | **Os04t0535600-02** | **Similar to H0502G05.3 protein** | **48.355** | **1.676** | **1.261** |
|  | **Os03t0772800-02** | **Cytochrome c oxidase, subunit VIa family protein** | **10.782** | **1.138** | **0.713** |
|  | **Os07t0262200-02** | **Similar to Prohibitin** | **31.954** | **0.997** | **0.686** |
|  | **Os08t0250200-01** | **ATPase, F1 complex, epsilon subunit, mitochondrial family protein** | **7.6707** | **0.946** | **0.764** |
|  | **Os03t0129900-01** | **NADH-ubiquinone oxidoreductase B18 subunit family protein** | **16.698** | **0.751** | **0.582** |
|  | Os03t0606200-01 | Mitochondrial ATP synthase 6 KD subunit | 6.9711 | 1.069 | 0.805 |
|  | **Os03t0774200-02** | **Similar to NADH-ubiquinone oxidoreductase subunit 8 (EC 1.6.5.3)** | **25.539** | **0.704** | **0.722** |
|  | Os08t0187800-01 | Similar to Glucose-6-phosphate/phosphate-translocator precursor | 42.018 | 1.023 | 0.664 |
|  | **Os02t0202400-01** | **ADP-glucose transporter, Plastidic translocator, Starch synthesis during seed development** | **45.363** | **1.051** | **0.777** |
| Other | **Os12t0485800-01** | **Prefoldin domain containing protein** | **16.446** | **0.757** | **0.993** |
|  | **Os07t0246200-02** | **Similar to Calreticulin (Fragment)** | **48.308** | **1.551** | **1.130** |
|  | **Os04t0107900-02** | **Heat shock protein 81-1 (HSP81-1) (Heat shock protein 83)** | **27.957** | **0.668** | **0.774** |
|  | **Os03t0780600-01** | **Tubulin beta-1 chain (Beta-1 tubulin)** | **49.822** | **1.440** | **0.954** |

Proteins indicated in bold represent those included in the gene/protein interaction network in Fig. 9.
